# Hippocampal spatial representations exhibit a hyperbolic geometry that expands with experience

**DOI:** 10.1038/s41593-022-01212-4

**Published:** 2022-12-29

**Authors:** Huanqiu Zhang, P. Dylan Rich, Albert K. Lee, Tatyana O. Sharpee

**Affiliations:** 1 Neurosciences Graduate Program, University of California, San Diego, La Jolla, CA USA; 2 Computational Neurobiology Laboratory, Salk Institute for Biological Studies, La Jolla, CA USA; 3 Princeton Neuroscience Institute, Princeton University, Princeton, NJ USA; 4 Howard Hughes Medical Institute, Janelia Research Campus, Ashburn, VA USA

**Keywords:** Learning and memory, Neural encoding

## Abstract

Daily experience suggests that we perceive distances near us linearly. However, the actual geometry of spatial representation in the brain is unknown. Here we report that neurons in the CA1 region of rat hippocampus that mediate spatial perception represent space according to a non-linear hyperbolic geometry. This geometry uses an exponential scale and yields greater positional information than a linear scale. We found that the size of the representation matches the optimal predictions for the number of CA1 neurons. The representations also dynamically expanded proportional to the logarithm of time that the animal spent exploring the environment, in correspondence with the maximal mutual information that can be received. The dynamic changes tracked even small variations due to changes in the running speed of the animal. These results demonstrate how neural circuits achieve efficient representations using dynamic hyperbolic geometry.

## Abstract (web summary)

Most spatial navigation studies assume a linear map of space. This study reports that neural representations of space follow non-linear hyperbolic coordinates. These representations also expand with experience along a maximally informative limit.

## Main

**Fig. 1:**
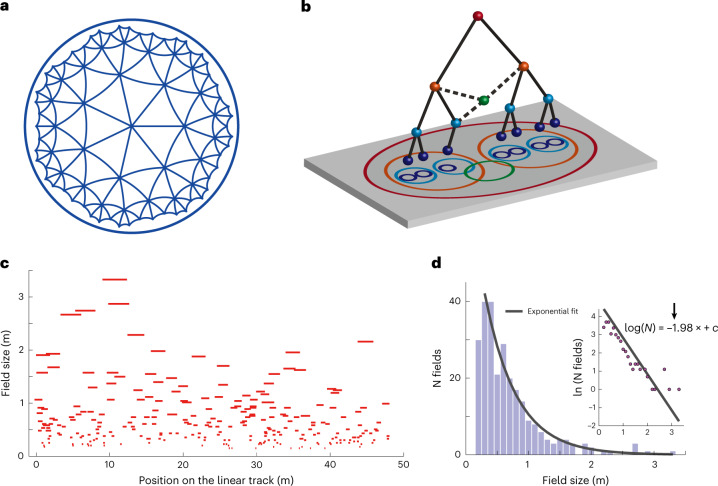
Construction of hierarchical organization of place cell responses that reflects underlying hyperbolic geometry. **a**, A Poincaré disk model of 2D hyperbolic geometry is shown for visualization of its similarity to a tree structure. Each curve represents the geodesic between the two connected points, and all triangles have the same size. **b**, Illustration of the construction of hierarchical representation from neuronal response properties. The tree structure does not have to be perfect to allow for mapping onto a hyperbolic geometry^14^. Some loops can be present (dashed lines) due to partial overlap between disks of neurons from different orders in the hierarchy. **c**, Place field size versus location of 264 place fields from 63 putative pyramidal cells from dorsal CA1 of a rat running on a 48-m-long linear track (Fig. 3b and Supplementary Fig. 1)^20^. **d**, Histogram of place field sizes shown in **c**. Gray line shows the maximum likelihood exponential fit. *P* value of χ^2^ GOF test is 0.851 (χ^2^ = 5.56 and d.f. = 10). Inset shows the same plot with log-scale on the *y* axis. The straight line shows the least-squares linear regression with slope forced to be the exponent of the exponential fit. Source data

Many aspects of our daily lives can be described using hierarchical systems. As we decide how to spend an afternoon, we may choose to read a book in a coffee shop or go to a shopping mall, which gives rise to a set of new questions: what coffee, which store and so forth, instantiating a decision tree^1,2^. Spatial navigation provides an example of decision-making in which hierarchical planning is useful^3^. Hierarchical organization in networks can provide several advantages, including achieving maximally informative representation of input signals^4^ and efficient routing of signals in cases where network links are subject to change^5^. The latter property is especially useful for neural networks where connections between neurons change over time. However, to realize these advantages, the networks should be organized hierarchically in such a way as to follow a hidden hyperbolic geometry^5^. Unlike Euclidean geometry, hyperbolic geometry is negatively curved. This results in an exponential expansion of volume with the distance from the center, perceptual compression of large distances and distortions in the shortest distance paths between points, which now curve toward the representation center (Fig. 1a)^6^. Thus, hyperbolic organization would go against the everyday intuition that the brain represents distances around us linearly.

We investigated whether hyperbolic geometry underlies neural networks by analyzing responses of sets of neurons from the dorsal CA1 region of the hippocampus. This region is considered essential for spatial representation^7–9^ and trajectory planning^10^. CA1 neurons respond at specific spatial locations, termed place fields^11^. We will first illustrate the main idea by describing how neurons can be organized into a hierarchical tree-like network using a simplified picture where each neuron has only one place field in an environment. We will then follow up with analyses that take into account the presence of multiple place fields per neuron^12^.

## Results

### Geometry of neural representation in CA1

The idea that hyperbolic geometry potentially underlies the neural representation of space follows from the construction illustrated in Fig. 1b. Each point in the two-dimensional (2D) plane represents an abstract neural response property, and a disk represents the collection of all the properties for one neuron in CA1. The more two disks overlap, the more similar the response properties of the corresponding two neurons are and, thus, the higher their response correlation. We now assign neurons that have larger disks in the plane to higher positions within the hierarchy (quantified by the *z* coordinate in the three-dimensional (3D) space) compared to neurons with smaller disks. The *x,y* coordinates are taken directly as the center positions of their disks. A link from a higher-to-lower-tier neuron is made if the larger disk contains (at least partially) the smaller disk. The resulting construction generates an approximate tree-like structure. The tree-like structure can, in turn, be viewed as a discrete mesh over the underlying hyperbolic geometry^13,14^. The leaves of the tree correspond to peripheral points, whereas roots of the tree correspond to positions closer to the origin of the hyperbolic geometry (Fig. 1a). We note that, in general, the plane can be of any dimension and is drawn here as 2D mainly for illustrative purposes. In the simplified case of each neuron having only one place field, the plane can be interpreted as physical space and the disks as being the place fields of the neurons. In this setting, the distribution of one-dimensional (1D) place field sizes should follow approximately an exponential distribution:

1 $$p\left( s \right) = \frac{{\zeta \sinh \left( {\zeta \left( {s_{\max } - s} \right)} \right)}}{{\cosh \left( {\zeta s_{\max }} \right) - 1}} \approx \zeta e^{ - \zeta s}$$

where *p*(*s*) is the probability density of place field sizes, and *s*_max_ is the maximal place field size. The exponent in this distribution is the curvature *ζ* of the representation (or, equivalently, the size of the hyperbolic geometry with unit curvature). In Fig. 1c,d, we indeed find that an exponential distribution fits the distribution of place field sizes well (*P* = 0.851, χ^2^ goodness-of-fit (GOF) test). A caveat with this analysis, however, is that, strictly speaking, it applies to the case where each neuron has one place field, whereas multiple place fields are observed per neuron in moderately large environments^12^.

**Fig. 2:**
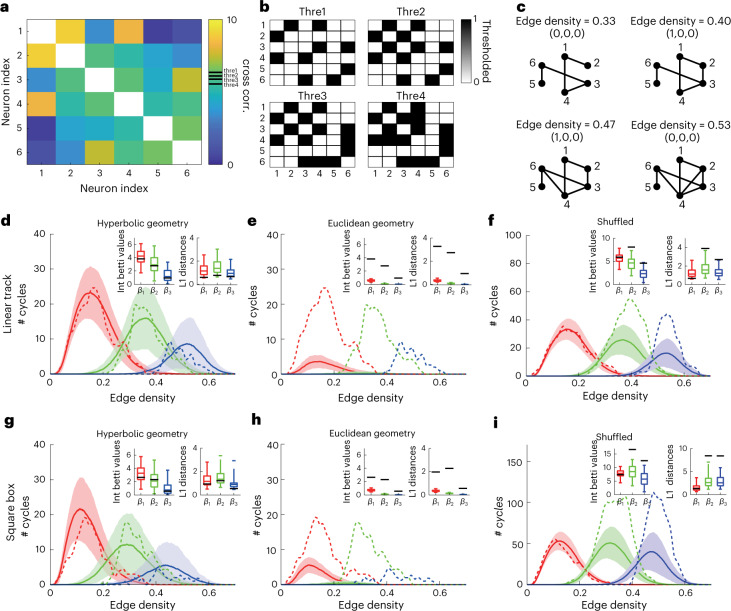
Neuronal activity in CA1 is topologically consistent with a 3D hyperbolic but not a Euclidean geometry. **a**–**c**, Illustration of the topological algorithm on an example correlation matrix. **a**, Example pairwise correlation matrix for six neurons. **b**, Correlation matrices after various thresholding. The threshold gradually decreases from top-left to bottom-right such that correlation between more pairs of neurons becomes significant. **c**, An edge connecting two nodes is formed when the corresponding entry in **b** is non-zero. Edge density measures the fraction of edges connected out of the total number possible. The numbers in each set of parentheses represent the number of 1D, 2D and 3D cycles (or holes) in the corresponding graph, respectively (note that the dimensionality of the cycles is not directly related to the dimensionality of the underlying geometry). This example demonstrates how a 1D cycle appears due to new edges being formed and then disappears because of formation of cliques. The number of such cycles across the entire range of edge densities (or thresholds) gives the Betti curves. **d**,**g**, Experimental Betti curves (dashed) for 1D (red), 2D (green) and 3D (blue) cycles are statistically indistinguishable from those generated by sampling (*n* = 300 replicates) from 3D hyperbolic geometry (solid line and shading indicate mean ± s.d.) for linear track exploration with radius = 15.5 (**d**) and square box exploration with radius = 11.5 (**g**). Insets show comparison of the curve integrals and the L1 distances from model Betti curves against those of the experimental Betti curves. Boxes show the model interquartile range. Upper and lower whiskers encompass 95% of the model range. Black lines indicate experimental values. **e**,**h**, Betti curves generated from 3D Euclidean geometry are statistically different from the experimental Betti curves in both linear (**e**) and square (**h**) environments. **f**,**i**, After shuffling spike trains, Betti curves generated from 3D hyperbolic geometry are no longer consistent with the experimental Betti curves in both linear (**f**) and square (**i**) environments. Session ID for square box: ec014.215. Source data

To quantitatively test whether hyperbolic geometry underlies neural representation in CA1, we employed a statistical tool from topology that does not directly rely on the computation of place fields. Instead, the method defines distances between neurons based on the pairwise correlations between their activities. This pairwise correlation is intuitively reflective of the degree of place field overlap between two neurons^15^ and is capable of accounting for neurons with multiple fields. These distances are then analyzed to determine if they produce the same topological signatures as points sampled from different geometries^16,17^. The topological signatures used by the methods are the so-called Betti curves^16^. These are obtained by varying the threshold for what constitutes a significant connection between neurons and counting cycles in the thereby generated graph (Fig. 2a–c). One of the advantages of this method is that, because it considers all possible values of the threshold, it is invariant under any non-linear monotonic transformations of the correlation values. Similar topological methods based on persistent homology have been used to study manifold structures of population responses in the head direction and grid cell systems^18,19^.

**Extended Data Fig. 1:**
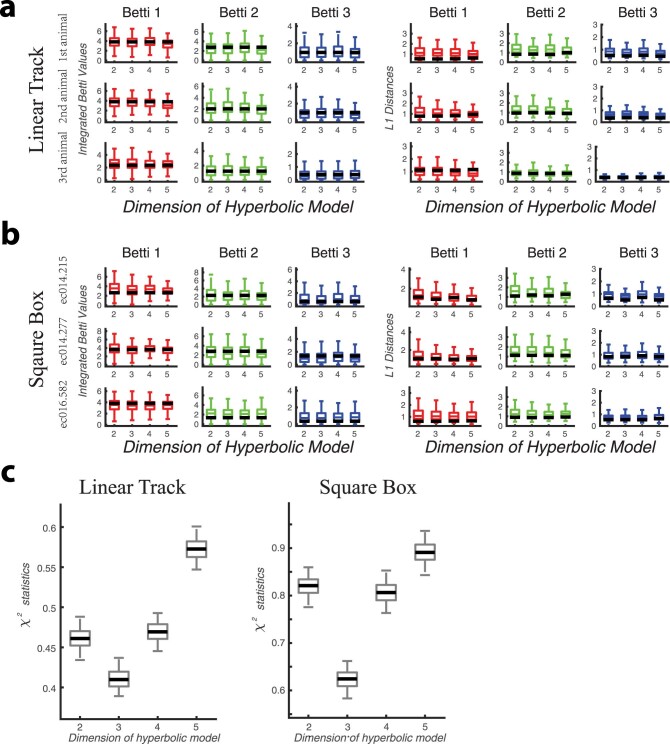
Betti curves generated from hyperbolic geometry of low dimensions (with optimized radius) are statistically indistinguishable from the experimental Betti curves for both 48m-linear track (a) and square box (b) environments with 3D being the optimal dimension in general. For each environment type, three sessions with the most number of neurons recorded are shown. Session IDs are shown on the left. The Betti curves are statistically similar both in terms of the area under the curve (measured by integrated Betti value) and the curve shape (measured by L1 distances between curves). Boxes show model interquartile range (n = 300 independent replicates). Center line indicates model median and upper and lower whiskers extend to the most extreme data points excluding outliers. Black lines indicate experimental values. (c) χ^2^ statistics (see Methods) are used to quantify how well the experimental Betti curves are fitted by model Betti curves from different dimensions. Results from two example sessions are shown, one for each environmental type (linear vs. square). Boxes show interquartile range (n = 500 bootstraps). Center line indicates median. Upper and lower whiskers encompass 95% of data. One-way ANOVA for each session: *p* < 10^−8^ across all dimensions (linear: F = 11239.87, square: F = 13613.23). Tukey’s post hoc test indicates that 3D fits are significantly better than fits from other dimensions (*p* < 10^−8^ for all 3 pairwise comparisons). Same conclusions hold for two-way ANOVA across all sessions in (a) and (b). Source data

**Extended Data Fig. 2:**
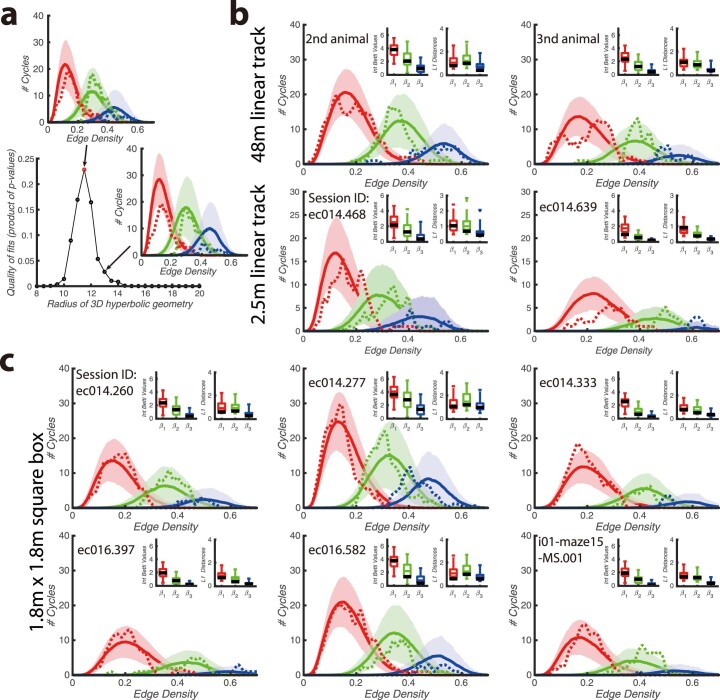
Betti curves generated from 3D hyperbolic geometry are statistically indistinguishable from the experimental Betti curves for both linear track and square box environments. **(a**) Illustration on how the hyperbolic radius is estimated for each session. 3D hyperbolic geometries of different radii are used to generate model Betti curves (solid lines and shading in the insets indicate mean ± std), which are then compared against the experimental Betti curves (dashed lines in the insets) of the session for *p*-values of integrated Betti values and L1 distances. The product of *p*-values versus radii are plotted and the radius of the session is estimated to be the radius that gives the largest product. (**b**) Linear track datasets. Notations are as in Fig. 2. Boxes show model interquartile range (n = 300 independent replicates). Center line indicates model median and upper and lower whiskers encompass 95% of model range. Black lines indicate experimental values. 48-m linear track datasets are from Rich, 2014. The rest are publicly available datasets collected by Dr. Buzsaki’s group including the two 2.5-m linear track datasets (2nd row in **b**) and the square box datasets (**c**). Session IDs are shown at the upper-left corner of each panel. See Supplementary Table 1 for all the radii used and the fitting statistics. Source data

**Extended Data Fig. 3:**
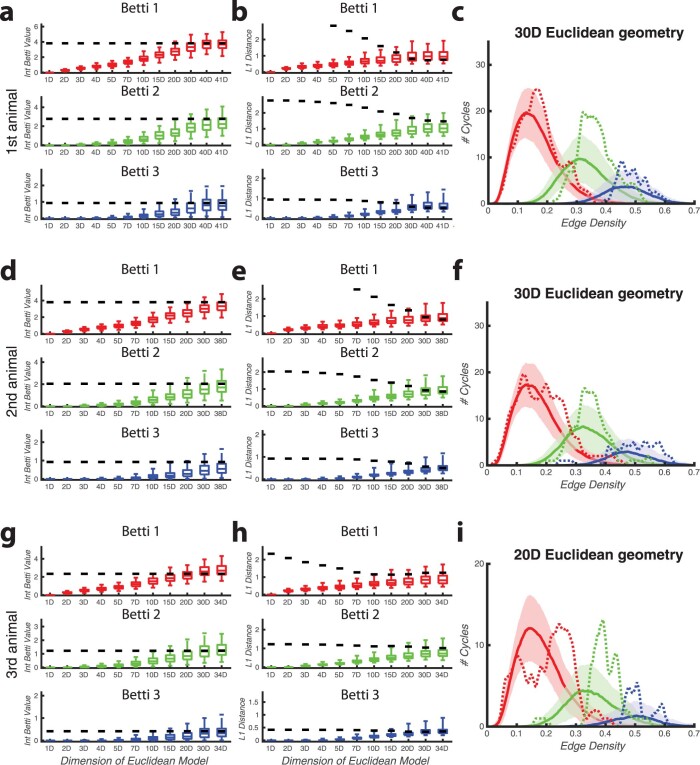
Betti curves generated from low-dimensional Euclidean geometries are statistically different from the experimental Betti curves in 48-m linear track exploration. Top to bottom rows represent different animals with N = 41, 38 and 34, respectively. (**a,d,g**) Distribution of experimental integrated Betti values (black lines) versus model statistics (boxplots). (**b,e,h**) Distribution of experimental L1 distances (black lines) versus model statistics (boxplots). Boxes show model interquartile range (n = 300 independent replicates). Center line indicates model median and upper and lower whiskers encompass 95% of model range. Black lines indicate experimental values. (**c,f,i**) Experimental Betti curves (dashed lines) and model Betti curves (solid lines and shading indicate mean ± std) from Euclidean geometry of dimension shown on the top. Source data

**Extended Data Fig. 4:**
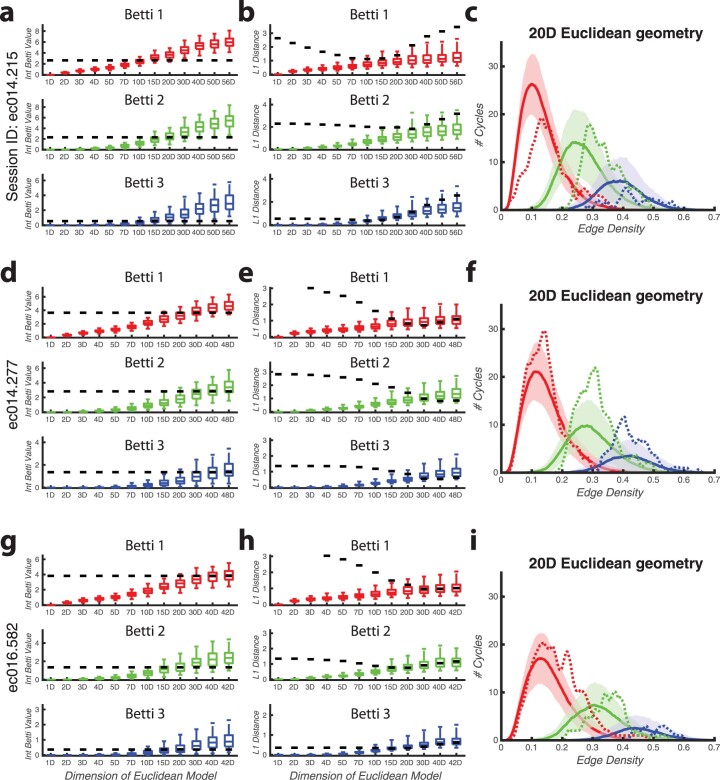
Betti curves generated from low-dimensional Euclidean geometries are statistically different from the experimental Betti curves in square box exploration. Top to bottom rows represent different datasets (session IDs shown on the left) with N = 56, 48 and 42, respectively. (**a,d,g**) Distribution of experimental integrated Betti values (black lines) versus model statistics (boxplots). (**b,e,h**) Distribution of experimental L1 distances (black lines) versus model statistics (boxplots). Boxes show model interquartile range (n = 300 independent replicates). Center line indicates model median and upper and lower whiskers encompass 95% of model range. Black lines indicate experimental values. (**c,f,i**) Experimental Betti curves (dashed lines) and model Betti curves (solid lines and shading indicate mean ± std) from Euclidean geometry of dimension shown on the top. Source data

We first applied this method to recordings of putative active pyramidal (average firing rate between 0.1 Hz and 7 Hz) cells (*n* = 113, average firing rate ± s.d. = 0.42 ± 0.35 Hz) from dorsal CA1 of three rats (one session per animal) running on a novel 48-m-long linear track^20^. We found that experimental Betti curves were not consistent with samples from a Euclidean geometry but were consistent with a 3D hyperbolic geometry (Fig. 2d,e). Statistical analyses showed that 3D hyperbolic geometry provided the overall best fit compared to other dimensionalities of the hyperbolic geometry (Extended Data Fig. 1; two-way ANOVA across sessions: *F* = 7,924.61, *P* < 10^−8^; Tukey’s post hoc test: *P* < 10^−8^ for all pairwise comparisons between 3D fits and fits from other dimensions). Similar low dimensionality of population activity in dorsal hippocampus has been reported with other manifold inference methods^21,22^. We note that three dimensions is also the expected dimensionality for a hierarchical set of 2D place fields according to the construction illustrated in Fig. 1b. The pairwise correlations were computed separately for each experimental session. Different sessions yielded latent 3D hyperbolic geometries of different radii (15.5, 15 and 13; see Methods and Extended Data Fig. 2a on how hyperbolic radius is estimated for each session). The same conclusions held for datasets of dorsal CA1 neurons recorded when rats explored 2.5-m linear tracks and 1.8 × 1.8-m square boxes for longer durations. We tested two (total number of putative active pyramidal neurons is 77, average firing rate ± s.d. = 0.92 ± 0.75 Hz) and seven (*n* = 274, average firing rate ± s.d. = 0.68 ± 0.61 Hz) publicly available datasets, respectively, for the above two environments, selecting recording sessions that had more than 50 neurons (not necessarily active) recorded simultaneously^23,24^. In each case, a 3D hyperbolic geometry produced Betti curves that matched those computed from data. In Fig. 2, we show one example analysis for each environment type (linear or square), and the rest of the datasets are shown in Extended Data Figs. 2, 3 and 4. The fitting statistics and the hyperbolic radii estimated for all sessions are summarized in Supplementary Table 1.

**Extended Data Fig. 5:**
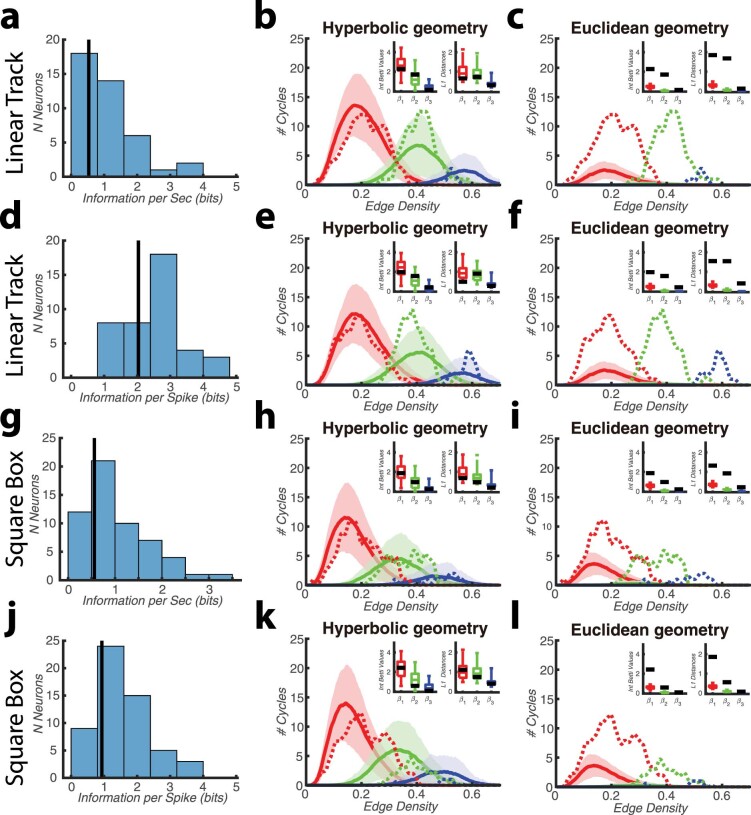
Betti curves generated from neurons with high spatial information are statistically indistinguishable from those of 3D hyperbolic geometry. (**a**) Distribution of information of each neuron’s response about the animal’s location per second. Black line indicates 25 percentile cutoff of the distribution. Those above the cutoff are used for Betti curve analyses in **b,c**. (**b,c**) Same notations as in Fig. 2. (**d**) Distribution of information of each neuron’s response about the animal’s location per spike. Black line indicates 25 percentile cutoff of the distribution. Those above the cutoff are used for Betti curve analyses in **e,f**. For **a–f**, the animal with the most number of active neurons recorded on the 48-m linear track is used (same one as in Fig. 2d-f). (**g–l**) Same analyses for a session in square box. Again, the session with the most number of active neurons is used (same one as in Fig. 2g-i, session ID: ec014.215). For all boxplots, boxes show model interquartile range (n = 300 independent replicates). Center line indicates model median and upper and lower whiskers encompass 95% of model range. Black lines indicate experimental values. Source data

**Extended Data Fig. 6:**
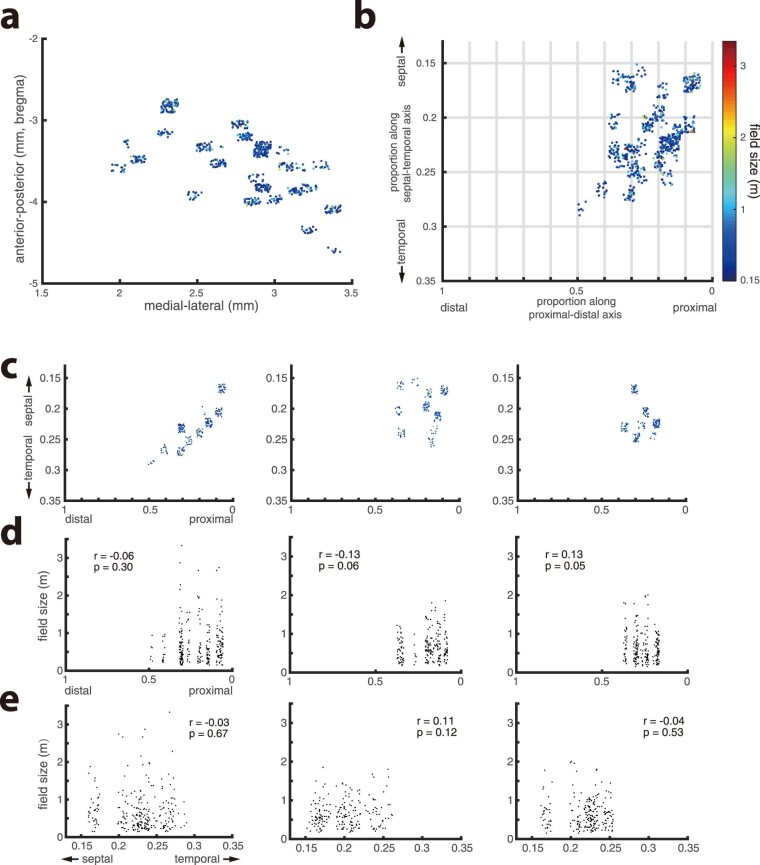
Place field size on the linear track as a function of anatomical recording location. (**a**) The location of each place field-corresponding cell viewed from above the septal half of CA1, with the field size denoted by the color scale at the right. (**b**) The same fields as in (**a**) but plotted in terms of the relative septal-temporal and proximal-distal coordinates of CA1. (**c**) As in (**b**) but separated by animal. (**d**) and (**e**) show the field sizes with respect to the two hippocampal axes, as well as the Pearson’s correlation coefficients and associated two-sided *p*-values. Jitter has been added to all anatomical values to improve visualization. Source data

As a control, we verified that shuffled spike trains (spike times of individual neurons were shifted by the same amount to preserve firing statistics) produced correlation matrices that were not consistent with a 3D hyperbolic geometry (Fig. 2f,i). Also, restricting our analyses to subsets of neurons with high spatial information yields the same results that 3D hyperbolic geometry, but not Euclidean geometry, underlies the neural representation (Extended Data Fig. 5). We note that the hierarchical organization we study works in addition to those observed across larger extents of the hippocampus and the entorhinal cortex^25–27^. This is because the variation of place field sizes determined from our data was not significantly correlated with the anatomical positions of the neurons recorded within the hippocampus (Extended Data Fig. 6), which, in our experiments, are largely confined to dorsal CA1, comprising only a fraction of the span of dorsal–ventral axis that was recorded in the above references^25,27^. These analyses demonstrate that neural activity in CA1 consistently conforms to a 3D hyperbolic geometry, with variations in the size of the hyperbolic geometry across environments, with values that range within 10.5−15.5 (in units of inverse curvature).

### Hyperbolic representation expands with experience and information acquired

**Fig. 3:**
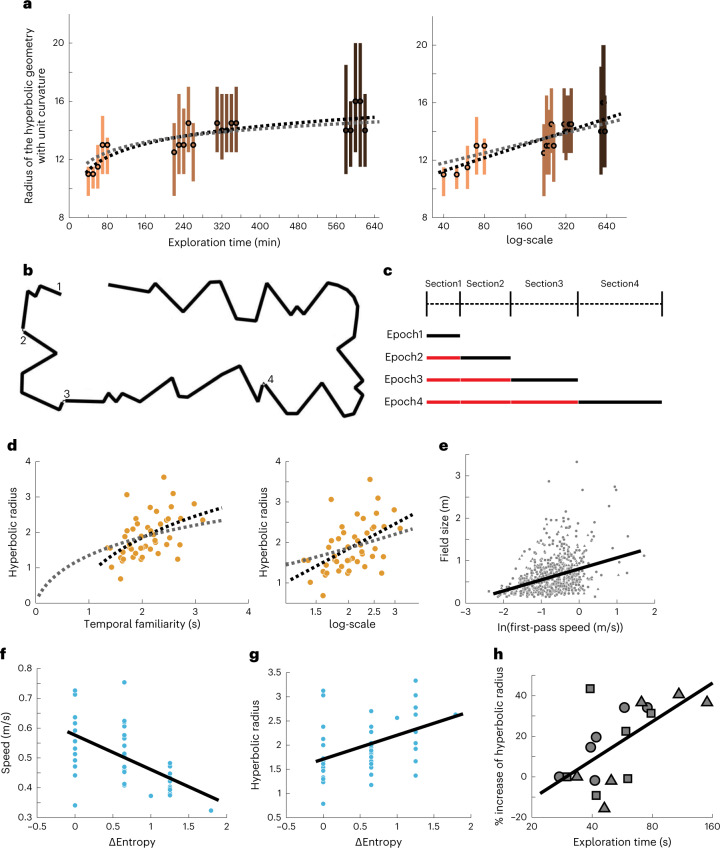
Hyperbolic geometry of neural representation expands with the logarithm of exploration time. **a**, Radius of the hyperbolic representation of square box is estimated using topological analysis. Dots represent median estimates, and lines represent 95% confidence interval. Dashed black line is the least-squares regression of data with log-scale on the *x* axis (*r* = 0.85, *P* = 3 × 10^−6^). Dashed gray line shows the least-squares fit of Eq. (2). **b**, Scale drawing of the 48-m linear track. The start of each track section is marked by numbers. **c**, Schematic of the experimental paradigm. During the first epoch, only the first section of the track is available to an animal. The linear track is progressively extended after each epoch. Total track length in the four epochs was 3, 10, 22 and 48 m. Black lines indicate periods when the animal is exposed to the corresponding section for the first time. Red lines indicate non-first exposures. **d**, Hyperbolic radius grows with temporal familiarity with the segments. Dashed black lines show the least-squares regression of data with log-scale on the *x* axis (*r* = 0.50, *P* = 0.0003), and dashed gray lines show the least-squares fit of Eq. (2). **e**, Field sizes increase with the animal’s first-pass speed through the field (*r* = 0.35, *P* = 9 × 10^−21^). Different symbols indicate fields of different animals. **f**, Animals run slower in segments of higher entropy (*r* = −0.56, *P* = 4 × 10^−5^). **g**, Radius of representation increases with entropy of the track (*r* = 0.42, *P* = 0.004). **h**, During subsequent exposures (red lines in **c**), hyperbolic radius increases with familiarity (*r* = 0.62, *P* = 0.0057). Symbols represent different animals, whose least familiar points are used as normalization. Each data point is an animal in one epoch-section period. Source data

**Extended Data Fig. 7:**
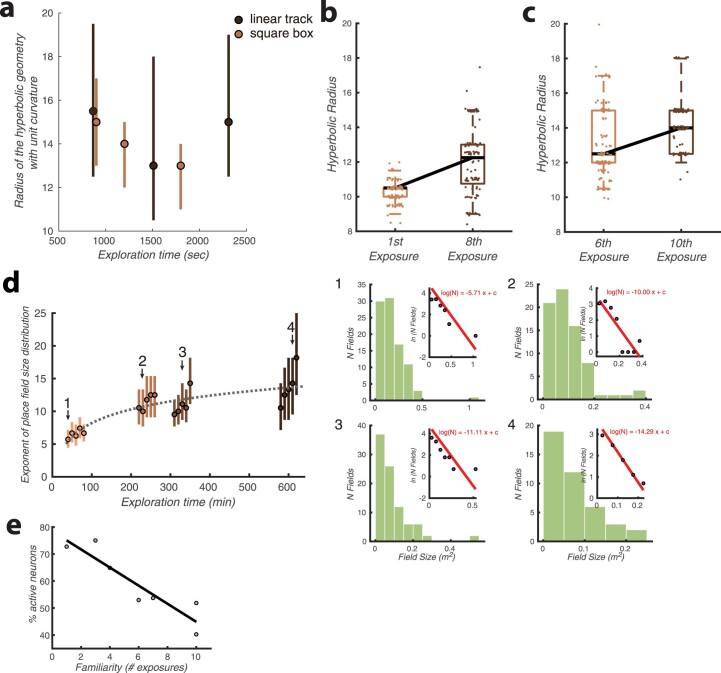
The hyperbolic representation sizes are similar initially across environments and change over time. (**a**) Estimated hyperbolic radii are similar for linear track and square box environments during the initial exposures. Dots represent median estimates (n = 100 independent sampling), lines represent 95% confidence interval. For linear track, same sessions used as in Fig. 2d and Extended Data Fig. 2b first row; square box session used: ec014.215. (**b**) Estimated radius increases for the same animal’s 1st and 8th exposure to a linear track environment. Session ID: ec014.468 and ec014.639. *p*-value = 4∙10^−15^ using two-sample *t*-test for same mean (two-sided). (**c**) Estimated radius increases for another animal’s 6th and 10th exposure to a square box environment. Session ID: ec016.397 and ec016.582. *p*-value = 1.3∙10^−5^ using two-sample *t*-test for same mean (two-sided). Hyperbolic radii are estimated using topological analyses for both **b** and **c**. For boxplots in **b** and **c**, boxes show interquartile range (n = 100 independent sampling). Center line indicates median and upper and lower whiskers encompass 95% of data. (**d**) Exponent of distribution of place field sizes increases over time (*r* = 0.85, *p* = 2∙10^−6^ with x-axis has log-scale). Same square box sessions used as in Fig. 3a. Dots and error bars (n = 100 independent sampling) indicate medians and 95% confidence intervals. Distribution of 4 sample 20-min sections indicated in the left panel are shown on the right. Same notation as in Fig. 1d. (**e**) % active neurons (firing frequency between 0.1 and 7 Hz) decays with familiarity with the environment (number of times the animal has been exposed to the environment). Each data point is a session. Straight line shows the least-squares fit. *r* = −0.9174, with associated two-sided *p* = 0.00361. Source data

**Extended Data Fig. 8:**
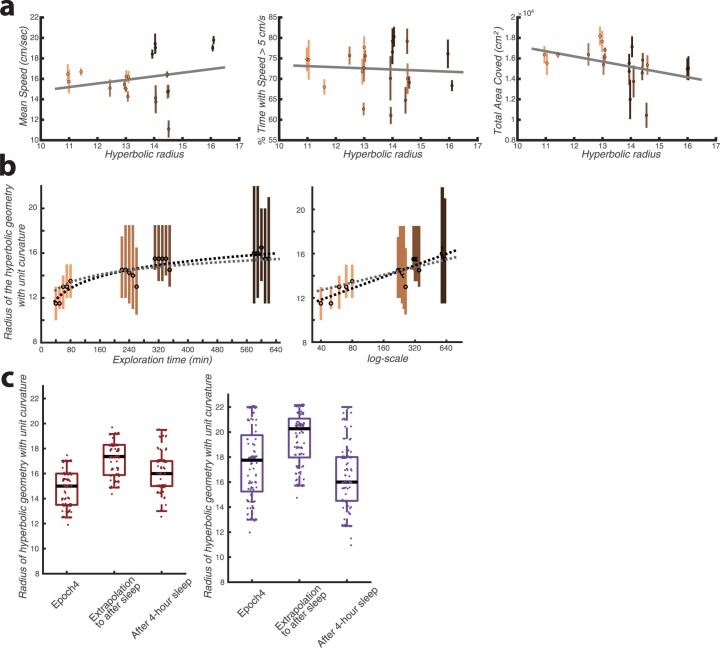
The increase of hyperbolic radius over time is independent of behavior, network state and time elapsed without experience. (**a**) No consistent change in the animal’s behavior is observed when the hyperbolic radius changes. Error bars represent standard deviations from jackknife resampling using 15 min out of 20 min (n = 4 jackknives). Same sessions used as in Fig. 3a. Gray lines show least-squares regression. Two-sided *p* > 0.05 for all three panels. Total area covered is calculated by breaking the square box into 2 × 2 cm grids and counting how many of the grids are passed by the animal during 15 min out of each of the 20-min intervals. (**b**) Hyperbolic radius increases over exploration time when periods with running speed <5 cm/sec were excluded from Betti curve calculation. Dots and error bars (n = 100 independent sampling) indicate medians and 95% confidence intervals. Dashed black line is the least-squares regression of data with log scale on the x-axis (r = 0.91, two-sided *p* = 2∙10^−8^). Dashed gray line shows the least-squares fit of Eq. 2. (**c**) Two animals are identified (one for each panel) which explored section 4 of the linear track during epoch 4 (Fig. 3c) for more than 15 min. They were put back into the sleep box for at least 4 h (at most 5 h) and then returned to the long track where they ran an additional 4–5 laps. Hyperbolic radii are estimated using the topological method for epoch 4 and after-sleep exploration. The middle boxplots are obtained by extrapolating hyperbolic radius during epoch 4 to after 4-h sleep using Eq. 2. For both animals, the actual hyperbolic radii estimated during exploration after 4-h sleep are significantly lower than the extrapolated values (two-sided *p* = 6∙10^−8^, 1.5∙10^−15^ for two animals, respectively, using two-sample *t*-test for same mean). Boxes show interquartile range (n = 100 independent sampling). Center line indicates median and upper and lower whiskers encompass 95% of data. Jitter has been added to the estimated hyperbolic radii to improve visualization. Source data

The environments in which these hyperbolic representations were observed differed in shape (linear versus square), size and also in the amount of time that the animals spent exploring them. First, we verified that the size of the hyperbolic representation was similar for data collected from linear and square environments, as long as the animals initially spent similar amounts of time in them (Extended Data Fig. 7a). Next, we investigated the effect of exploration time on the size of the resulting hyperbolic representation of the same environment that was initially novel to the animal. We found that the size of the hyperbolic representation was larger when the animal was more familiar with the environment, using the above topological method. The size increased with the logarithm of time that the animal had to explore it (Fig. 3a and Extended Data Fig. 7b,c). This is interesting because the logarithm of time is approximately proportional to the maximal amount of information the animal can acquire from the novel environment (considering an animal receiving a distinct combination of stimuli at each time step)^28^, with the exact relationship given by:

2 $$I = \log \left( {1 + \frac{T}{{t_0}}} \right) + \frac{T}{{t_0}}\log \left( {1 + \frac{{t_0}}{T}} \right)$$

where *t*_0_ is a constant that represents the product of sampling interval and the ratio between the effective signal *S*_*n*_ and noise *N*_*n*_ variances. In Fig. 3a, we show that this relationship accounts well for the expansion of the CA1 hyperbolic representations as the animal is exploring the environment. For these analyses, we sorted the datasets according to the number of times the animal had a chance to explore the square box; we also analyzed separately data from individual exposures (indicated by different colors in Fig. 3a) to the box in 20-min sections for their hyperbolic radii. We note that, although CA1 neural representations undergo substantial dynamic changes over these hour-long time scales of exposures across days^29,30^, the neural responses continued to be described by a 3D hyperbolic geometry with a systematic increase in its size. We verified that this was not due to changes in the animal’s locomotion and behavioral states; no significant correlation between representation size and parameters, such as the average running speed and active exploration time, was observed (Extended Data Fig. 8a). Excluding time periods with speed <5 cm s^−1^ also do not change the results (Extended Data Fig. 8b). We also verified that time elapsed without exploration (that is, outside the environment) cannot account for the expansion of representation observed (Extended Data Fig. 8c).

**Extended Data Fig. 9:**
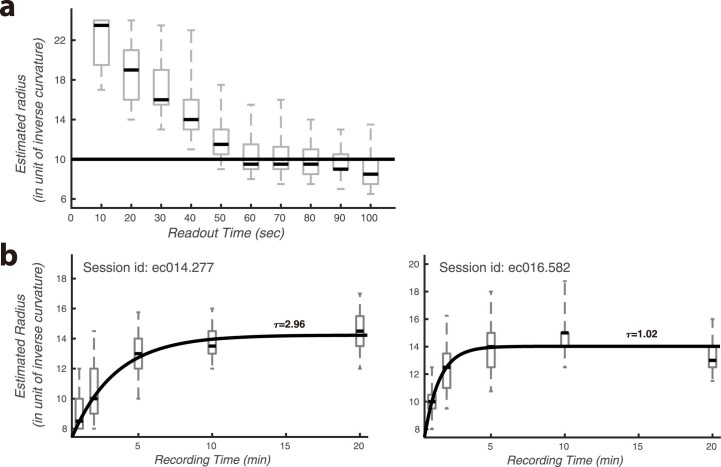
Bias exist when hyperbolic radius is estimated using Betti curve analyses from short neural recordings. (**a**) The estimated hyperbolic radius exhibits strong positive bias when the recording time is less than 1 min in a simulation (see Methods for the simulation details.). Black line at y = 10 represents the ground truth radius value used in the simulation. Boxes show interquartile range (n = 100 independent sampling). Center line indicates median. Upper and lower whiskers encompass 95% confidence interval of the radius estimate. (**b**) Hyperbolic radius is estimated from actual neural recordings of different length. Shown here are results for two example sessions (session IDs are given on the top left). Boxes show interquartile range (n = 100 independent sampling). Center line indicates median. Upper and lower whiskers encompass 95% of the radius estimate. An exponential function is then fitted to the medians with the time constant denoted on the plot. Source data

**Extended Data Fig. 10:**
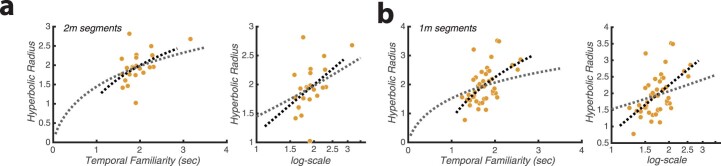
The radius of hyperbolic representation increases over timescale of seconds independent of segment length and exposures used in measure of familiarity. (**a**) Hyperbolic radius increases over time when 2-m segments are used instead of 1-m segments (*r* = 0.48, *p* = 0.02). (**b**) Hyperbolic radius increases over time when mean speed of all passes are used instead of first passes (*r* = 0.54, *p* = 8∙10^−5^). Source data

The same relationship between the size of the hyperbolic representation and exploration time was observed on shorter time scales, including the first seconds during initial exploration of a novel environment. For these analyses, we used the dataset from the 48-m-long linear track (Fig. 3b). The track was initially novel to the animals. In each epoch, an animal traversed the current total length of the available track 3–5 times. Between epochs, it was confined to the original start location while the track was extended (Fig. 3c). We first tested the relationship during initial formation of place fields by considering only periods when the animal explored the additional novel sections of the track introduced in each epoch (Fig. 3c, black lines). We estimated hyperbolic radii for each 1-m segment of the track by examining the distribution of place field sizes within it. In this situation, we are close to the one-field-per-neuron case for each 1-m segment (93.4% ± 6.6% place fields are from different neurons) and, thus, estimated the hyperbolic radii by examining the exponent of place field size distribution in Eq. (1) (Fig. 1d, arrow). We did not use the topological analysis here, because, with short recording duration, the method produces biased estimates of the radius, with larger bias for shorter recordings (Extended Data Fig. 9). We found that the hyperbolic radius increased with temporal familiarity with the environment (Fig. 3d). The temporal familiarity was calculated as the inverse of the mean speed (Methods). The same results were obtained when speed was computed for the initial traversal or across multiple traversals or when using a different segment length (Extended Data Fig. 10). Also, a significant positive correlation between field size and its first-pass speed was observed (Fig. 3e). Therefore, higher speed and, thus, less familiarity with the nearby environment produced larger place fields. This, in turn, led to a smaller hyperbolic radius or the exponent in Eq. (1).

We also tested the relationship among the radius of the representation, the speed of the animal and the information content of the environment. The shape of the environment is a key aspect of spatial information that can be perceived by the animal. For a linear track, the shape is determined by its angles. In our experiment, some 1-m segments had more turns, whereas others were mostly straight (Fig. 3b). One can quantify the additional information provided by the changes in trajectory of the track by computing the probability distribution of the change in angle *δθ* between segments and using the standard formula for entropy^31^. With this definition, straight segments have zero additional entropy, with larger values for more curved and varied segments. We found that animal speed was inversely related to the additional information (Fig. 3f), with lower speed in more informative segments. The more informative segments also generated representations with larger hyperbolic radius (Fig. 3g). However, the differences in the hyperbolic radius can be fully explained by differences in the time that the animal spent in a given segment. This is because, once controled for speed, representation size was no longer significantly correlated with additional information (linear partial correlation coefficient = 0.18, *P* = 0.236). We then focused on subsequent exposures to sections of the track (Fig. 3c, red lines). Similarly to the above observations, we found that the radius of the representation continued to increase with additional familiarity using the same measure of hyperbolic radius (Fig. 3h). These analyses indicate that the neural representation maintains hyperbolic organization after experience-induced re-organization with systematic increases in the size of the hyperbolic geometry.

### Observed hyperbolic representation maximizes spatial information given the number of CA1 neurons

**Fig. 4:**
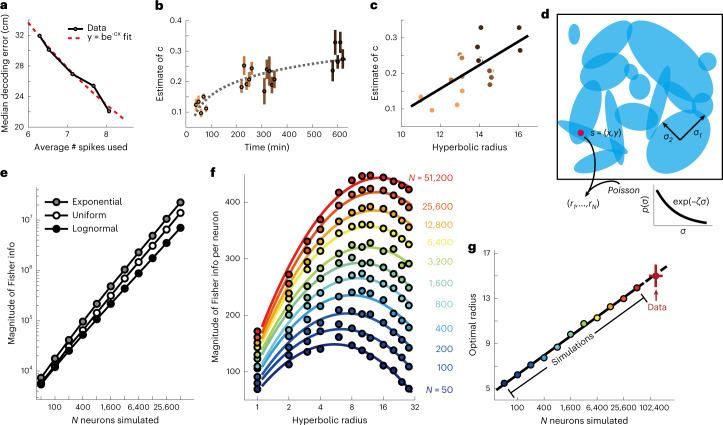
Increase in size of the hyperbolic representation results in higher accuracy in spatial localization. **a**, A Bayesian decoder with integration time window of *δt* = 500 ms is used for different number of subsampled neurons for each session (Methods). An example relationship is shown here, which is fitted with an exponential function of the form indicated to determine the value of *c* for each session. **b**, Estimates of *c* increase over time. Error bar represents the standard error of the estimate. Dashed gray line shows the least-squares fit with log-scale on the *x* axis (*r* = 0.86, *P* = 10^−6^). **c**, Estimates of *c* correlate significantly with hyperbolic radius (*r* = 0.70, *P* = 0.0007). Black line shows the least-squares fit. Jitter has been added to *x* values to improve visualization. **d**, Schematic of the modeling framework for computing Fisher information for how accurately an animal’s position can be decoded from neural responses: place fields are modeled as 2D Gaussian functions^47^, and neural response has Poisson variability^48^. **e**, Networks with exponentially distributed place field size provide more information about the animal’s position than networks where place field size is uniformly distributed (with the same mean size as the exponential distribution) or log-normally distributed (with the same mean and variance as the exponential distribution). *P* < 0.001 for equal means between information from exponential and uniform distributions, or exponential and log-normal distributions, at all number of neurons, using an unpaired two-sample *t*-test. **f**, Fisher information per neuron for networks of different sizes and of hyperbolic representations of different radii. **g**, Optimal hyperbolic radius depends logarithmically on the number of neurons in a network (with dashed portion showing extrapolation of the relationship). The extrapolated value for the full CA1 circuit agrees with median values (upper and lower endpoints of the cross) of hyperbolic radius determined in the last exposure of the square box in Fig. 3a. Left and right endpoints indicate range of experimental values 320,000 × 30% and 490,000 × 40%, respectively, for the number of active CA1 neurons. Source data

**Fig. 5:**
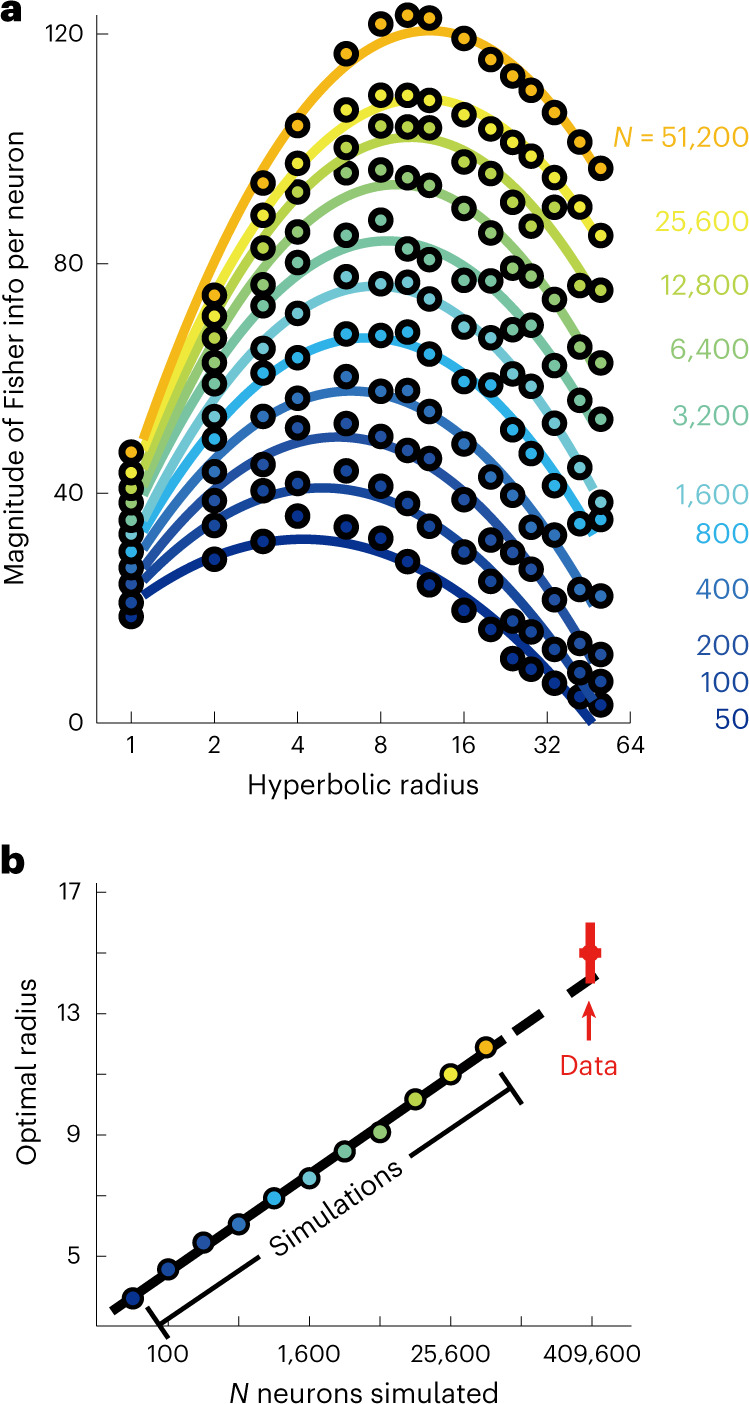
Theoretically optimal representation radius is consistent with the experimental value when each cell can have potentially multiple place fields. In this simulation, the statistics of number of place fields per cell are matched to the experimental values of sessions shown in Fig. 3 (mean = 0.98 and s.d. = 1.10, which determines the gamma distribution that, in general, describes number of place fields per cell^20^), and the size of the environment is matched to the experimental value of 1.8 × 1.8 m. **a**, Fisher information per neuron for networks of different sizes and of hyperbolic representations of different radii. **b**, The extrapolated value for the full CA1 circuit agrees with median values of radius determined in the last exposure of Fig. 3. Same notation as in Fig. 4g, except left and right endpoints of the cross indicate range of experimental values 320,000 and 490,000, respectively, for the number of all CA1 neurons, as the gamma distribution is used to capture number of place fields for all neurons as opposed to only active neurons. Source data

To quantify how well the CA1 neurons could support spatial localization, we computed Bayesian maximum likelihood decoder for the animal’s location based on neural responses from the square box datasets. To avoid the potential confound associated with differences in spike rates across sessions, we subsampled, for each session, subsets of neurons resulting in different overall spike rates and estimated how much the median decoding error decreases with increasing the number of spikes used for decoding. The decoding error decreased exponentially with the number of spikes (Fig. 4a). The exponent *c* in this case indicates how efficient an extra spike is in reducing decoding error. Similarly to the radius of hyperbolic representation, we found that *c* increased consistently over time (Fig. 4b). Figure 4c shows that exponent *c* also correlated significantly with hyperbolic radius. To establish a causal relationship between representation radius and accuracy of spatial localization, we computed the Fisher information, a measure of read-out accuracy^32,33^, for populations of model neurons with place field sizes distributed according to different distributions (Fig. 4d). In the one-field-per-neuron case, we found that populations of neurons whose place field sizes were exponentially distributed, as expected for a hidden hyperbolic geometry, provided more accurate spatial decoding compared to the cases of uniformly or log-normally distributed place field sizes (Fig. 4e and Supplementary Fig. 2). Furthermore, for a given network size, there was an optimal size of the hyperbolic representation that maximizes Fisher information (Fig. 4f). The optimal value is determined as a tradeoff between two factors. On the one hand, larger representations include exponentially more small place fields and have higher capacity to represent space. However, they also require more neurons for their sampling and can become undersampled if the number of neurons is limited. The interplay between these two factors determines the optimal size of the representation for a given number of neurons. The simulations indicate that the optimal representation size increases with the logarithm of the number of neurons (Fig. 4g). This theoretical prediction can be tested against data on the number of active neurons in the CA1 region. The CA1 region of rat hippocampus contains roughly 320,000–490,000 pyramidal neurons^34,35^ with about 30–40% of neurons being active in any given environment^29,36,37^ (Extended Data Fig. 7e). Extrapolating the theoretical curve in Fig. 4g to this number of neurons, we found a close match to the representation size extracted from the analysis of correlation in neural responses in square box (Fig. 3a). In any moderately large environment, however, individual CA1 neurons can have multiple place fields^12,20,38,39^. Therefore, we also simulated the case where each cell can have multiple place fields according to a gamma-Poisson model^20^ with statistics matched to the experimental data, and we found that the same conclusions hold (Fig. 5). This match indicates that the hierarchical arrangement of neural responses is well-matched to the overall number of neurons and their propensities for forming fields.

### Exponential distribution of place field sizes

**Fig. 6:**
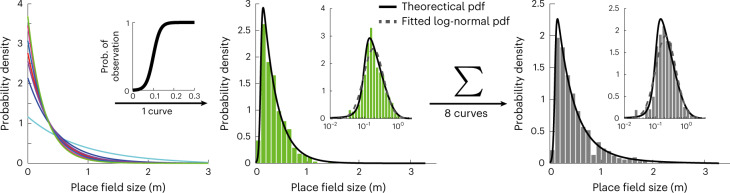
Illustration of observation of a log-normal distribution of place field sizes. Random samples from each of eight different sinh distributions (left) are drawn. Suppressing the probability of observing small place fields according to a sigmoidal function (left inset) leads to a distribution that approximates log-normal (middle). The middle panel shows the resulting observed histogram for one sinh distribution. Inset shows log-scale on the *x* axis. When sample size is not too large (*n* = 800 in this simulation), the histogram can be fitted by a log-normal distribution with *P* = 0.07 (χ^2^ GOF test, χ^2^ = 11.66 and d.f. = 6). Black curve represents the theoretical density function, and gray line represents the fitted log-normal distribution. The right panel shows the results after pooling from eight distributions where an even better fit with log-normal can be obtained (*P* = 0.43, χ^2^ GOF test, χ^2^ = 4.87 and d.f. = 5) at a moderate sample size (*n* = 800 in this simulation). pdf, probability density function. Source data

So far, we focused on using an exponential function to analyze the distribution of neural place fields, because this distribution connects with hyperbolic representations and makes it possible to infer its curvature. However, other skewed distributions, in particular the log-normal distributions, have similar properties at large values and have been shown to match neural data^40^. Therefore, it is worthwhile to discuss factors that distinguish and relate both model distributions to each other. First, it is important to note that these two distributions differ most for small place field sizes, and these are the place field sizes that are most affected by biases in the current experimental recording and place field determination methods. Very small place fields are often discarded by smoothing procedures used to estimate neural place fields or even by default (note their absence in Fig. 1c)^40^. Figure 6 presents simulations showing that this sampling bias alone can transform an exponential distribution of place field sizes into an almost log-normal one. Therefore, previous demonstrations of log-normal distributions of place field sizes do not necessarily argue against the presence of exponential distributions. Second, as we have observed, the curvature of hyperbolic representations (or, equivalently, their radius relative to unit curvature) varies across environments depending on the animal exploration time (Fig. 3). Averaging of exponential distributions of place field sizes with different exponents will further bring the resultant distribution closer to the log-normal family of curves (Fig. 6, right panel). This effect will be even stronger when data collected from different animals and environments are pooled together as more different distributions are summed together.

## Discussion

**Fig. 7:**
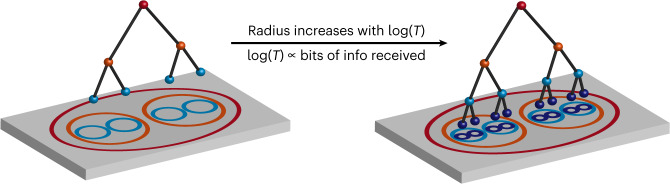
Schematic illustration of how increased hyperbolic radius affects neural representation of space. As radius grows over time, exponential distribution of place field sizes shifts toward smaller place fields (Extended Data Fig. 7d) or larger depth in a discrete tree structure (Eq. 1), to improve spatial localization.

Several previous studies described how place fields re-organize with experience^29,30,41,42^. Here we describe that these changes not only include the emergence of fine-grained place fields but also result in coordinated changes across the network. It turns out that the logarithmic growth of the hyperbolic radius *R* ~ log(*T*) that we observe here can be explained by a simple mechanistic model where place fields of a small size appear as soon as the animal spends a certain minimal required time *t*_0_ within the field (Fig. 7). Because small place fields map to the edge of the hyperbolic geometry, their number *N* increases exponentially with the radius *R*. Thus, the hyperbolic representation will approximately reach radius *R* once the total exploration time *T* reaches *T* = *Nt*_0_ ~ exp(*R*)*t*_0_, or:

3 $$\log \left( {T/t_0} \right) = R$$

as observed experimentally (Fig. 3). The logarithmic growth of *R* with time in turn matches the entropy that can be acquired from the environment (Eq. (2)). These arguments demonstrate that it is possible to achieve efficient representations from the information theory point of view using simple mechanistic rules for the addition of small place fields (Eq. (3)). The constant *t*_0_ now acquires multiple interpretations. In the mechanistic model of place field addition and expansion of hyperbolic representation (Eq. (3)), *t*_0_ represents the minimal time required to form a place field. In the information acquisition formula, Eq. (2), *t*_0_ represents the temporal sampling interval. Being the only parameter in the information acquisition equation, *t*_0_ also determines the transition when the initially novel environment becomes familiar: during the initial exploration, the information and the hyperbolic radius both increase approximately linearly with time. For times much longer than *t*_0_, the linear increase changes over to a logarithmic one. The increase in the relative proportion of small place fields over time (Fig. 7 and Extended Data Fig. 7d) may also be achieved as a result of the overall decrease of firing rate in CA1 with familiarity^41,43^.

The hyperbolic representation was recently demonstrated for the olfactory system^17,44^. However, those studies analyzed only natural olfactory stimuli^17,44^ or human perceptual responses^17^. This left open the question of whether neural responses also have hyperbolic representation. We address this question here, not in the same sensory modality but for the more general hippocampal representation that interacts with many sensory modalities.

Hyperbolic representation describes a specific instantiation of hierarchical organization that offers many functional advantages^45^. One particularly important advantage for neural circuits is that it allows effective communication within the network using only local knowledge of the network and in situations where network links are changing in time^5^. We show here that hyperbolic representations also support more accurate spatial localization (Figs. 4 and 5). Related to this, recent literature indicates the presence of multi-scale place fields within dorsal CA1, which could improve spatial decoding especially in large environments^38,39^. The presence of hyperbolic geometry could be further supported by hierarchical representation across broader scales in hippocampus and entorhinal cortex^25–27^.

In addition to predictions for the spatial distribution of place field sizes, the hyperbolic representation also makes predictions for the temporal aspects of the neural code. The reason for this is that, within hyperbolic representations, addition is not commutative, meaning that the order in which vectors are added matters. For neural systems, this implies that the order in which spikes are received from different neurons should matter. A detailed investigation of these results comprises a promising direction for future research.

To summarize, we have shown that organizing neural circuits to follow latent hyperbolic geometry makes it possible to implement coherent large-scale reorganization. For example, this could be achieved by adjusting neuronal thresholds to control place field sizes (Fig. 7 and Extended Data Fig. 7d). These changes allow a continuous increase in representational capacity (Figs. 4 and 5). As such, this type of organization might provide a general principle underlying large structural changes of neural representations to benefit communication between brain areas, as has been observed, for example, in the parietal cortex^46^.

## Methods

### Data and processing

The dataset in which the rat was exposed to a 48-m linear track was recorded from five different animals along the entire track (see original paper^20^ for detailed procedure of surgery, training, recording, etc.). All procedures were performed according to the Janelia Research Campus Institutional Animal Care and Use Committee guidelines on animal welfare (protocol 11–73). In brief, the subjects were five adult male Long-Evans rats, 400–500 g at the time of surgery. Two rats had fewer than 30 active neurons (overall average firing rate within the range 0.1–7 Hz) recorded, and they were excluded from further analyses. The track and room were both entirely novel to the animals. Animals made 3–5 traversals of the full extent of the track during each epoch (Fig. 3b,c) while their neural activity was recorded using a 64-channel system. Spikes were detected by a 60–70-µV negative threshold on a 600–6,000-Hz filtered signal, and waveforms (32 samples at 32 kHz) were captured around each threshold crossing. Local field potential (LFP) (0.1–9,000 Hz) was recorded continuously at 32 kHz. The position of the animals was reconstructed from video taken by three wide-angle overhead cameras synchronized to the time stamp clock from the acquisition system using a time stamp video titler. Exponential fitting of place field size distribution is done with a maximum likelihood estimate. To take into account that small place fields tend to be undersampled, we explicitly excluded the smallest place fields (<25 cm, first column of histogram in Fig. 1d) and modified the prior accordingly. In this way, the presence or absence of these fields does not affect the estimation of curvature. For computation of the entropy of each segment of the linear track, turn angles were considered in each segment (with angles = 0 for straight segments). All turn angles were discretized into 15 bins, and the entropy for each segment was calculated as $\mathop {\sum}\nolimits_i {p_i\log _2\frac{1}{{p_i}}}$, where *i* indexes bins of the turning angles.

The first-pass speed for each place field was computed by dividing the field size by the first-time pass duration of the animal through the place field with the cell firing at least one spike (this ≥1-spike requirement is assumed for all of the following description of first-pass duration). The average speed in each 40-inch (~1-m) segment during place field formation was calculated as follows: average segment speed = (∑_i_l_i_)/(∑_i_ first-pass duration), where *i* is the index of place fields that were centered within the segment, and *l*_*i*_ is the size of each place field. We can then compute the temporal familiarity of the rat with the segment during place field formation as the time spent per unit length: temporal familiarity (s) = 1 m/average segment speed, which are used in Fig. 3d as the *x* coordinates. When segment length is not unit (as in Extended Data Fig. 10a), a normalization of segment length is applied to make the familiarity measures comparable.

For locating the neurons recorded in CA1, either immediately after the experiment or within a few days of it, 3–4 tetrodes were electrolytically lesioned (20 µA for 10 seconds) as fiducials, and animals were transcardially perfused with PBS, followed by a 4% paraformaldehyde solution. Brains were cut in 50-µm sections and stained with cresyl violet. Fiducial lesions, electrode tracks and the relative locations of the tetrode guide cannulas in each microdrive, as well as allowance for brain shrinkage, were used to estimate the AP and ML coordinates of each tetrode with respect to a rat brain atlas^49^. Only tetrodes localized to the CA1 region were used in analysis. The atlas was used to construct a 3D model of the CA1 pyramidal cell layer, allowing an estimate of the tetrode locations with respect to the septotemporal and proximodistal axes of CA1.

All the other datasets were obtained from http://crcns.org/data-sets/hc/hc-3, contributed by the Buzsáki laboratory at New York University^23,24^. Neural activity in these datasets was recorded using either four or eight shank probes at 20 kHz. Each shank has eight recording sites, making 32 or 64 possible recording sites (channels). Spike detection was performed on the output from raw data with an 800–5,000-Hz bandpass filtering. See http://crcns.org/files/data/hc3/crcns-hc3-processing-flowchart.pdf for more details about recording and experiments.

For rats exploring a 180 × 180-cm box, all sessions that have more than 50 simultaneously recorded CA1 neurons were included for analysis. We also excluded neurons that are marked as inhibitory or not identified and those that have average firing rates outside the range 0.1–7 Hz during the entire recording session. We also excluded neurons that have average firing rates between 0.1 Hz and 7 Hz but remain silent (fire zero spikes) for more than 30 min for potential death of the cell or movement of electrodes. Details of sessions used are summarized in Supplementary Table 2.

### Place field determination and spatial information calculation

For place field determination on the linear track, animals were allowed to make 3–5 traversals of the full extent of the track in four epochs^20^. Rate maps were constructed by taking the number of spikes in each 1-cm spatial bin of the track divided by the occupancy in that bin, for each of the two directions of movement. Both were smoothed with a Gaussian kernel with an s.d. of 10 cm. Only periods when the animal’s velocity was greater than 5 cm s^−1^ were included in the spatial firing rate maps. A place field was defined as at least 15 contiguous centimeters of the rate map in which the firing rate exceeded 2 Hz. Because, in linear tracks, place fields may be directional, place fields were detected independently for each directional firing rate map, outbound and inbound, and then fields in different directions were merged if either field showed at least 50% overlap with the other (for more details on experimental procedure, see ref. ^20^). For place field determination in Fig. 3h, we did not impose the 15-cm length requirement and did not merge place fields with different directions to have numerically more place fields and small place fields for estimating hyperbolic radius.

For place field determination in the square box of size 180 × 180 cm, rate maps were constructed by taking the number of spikes in each 2 × 2-cm spatial bin of the box divided by the occupancy in that bin. The rate map is then smoothed with a 2D Gaussian kernel with an s.d. of 10 cm. Again, only periods when the animal’s velocity was greater than 5 cm s^−1^ were included in the spatial firing rate maps. We then detected potential place fields as all contiguous regions in the rate map in which the firing rate exceeded 2 Hz, followed by a manual breakdown of some of these place fields into smaller ones when they have more than one peak in the firing rate map.

For spatial information calculation, the following formula was used^50^:

4 $${{{\mathrm{Information}}}}\,{{{\mathrm{per}}}}\,{{{\mathrm{spike}}}} = \mathop {\sum}\nolimits_{i = 1}^N {p_i} \frac{{\lambda _i}}{\lambda }\log _2\frac{{\lambda _i}}{\lambda }$$

5 $${{{\mathrm{Information}}}}\,{{{\mathrm{per}}}}\,{{{\mathrm{second}}}} = \mathop {\sum}\nolimits_{i = 1}^N {p_i} \lambda _i\log _2\frac{{\lambda _i}}{\lambda }$$

where *i* = 1, · · ·; *N* are pixel indices in the rate map; *p*_*i*_ is the probability of occupancy of pixel *i*; *λ*_*i*_ is the mean firing rate of pixel *i*; and *λ* is the overall mean firing rate of the cell on the whole track or box.

### Position decoding with a maximum likelihood decoder

The spatial firing rate maps are computed with the procedure described above except that we now use a 5 × 5-cm spatial bin instead of 2 × 2 cm. The time window we use is *δt* = 500 ms. Windows with mean speed less than 5 cm s^−1^ were not used for decoding. For each time window, we compute the log-likelihood of observing the spike count vector $\left\{ {n_j} \right\}_{j = 1}^N$ at each pixel *i* assuming conditional independence among neural responses:

6 $$\begin{array}{*{20}{l}}{\log p\left( {\left\{ {n_j} \right\}_{j = 1}^N|i} \right)}{ \,=\, \log \left( {\mathop {\prod}\nolimits_{j = 1}^N {p\left( {n_j|i} \right)}} \right)}\\ \qquad\qquad\qquad\quad\,\,{=\, \log \left( {\mathop {\prod}\nolimits_{j = 1}^N {Poisson\left( {n_j;\lambda _{ij} \ast \delta _t} \right)}} \right)}\\ \qquad\qquad\qquad\quad\,\,{=\, \mathop {\sum}\nolimits_{j = 1}^N {\log \left( {Poisson\left( {n_j;\lambda _{ij} \ast \delta _t} \right)} \right)}}\end{array}$$

where *λ*_*ij*_ is the mean firing rate of the *j*th neuron in pixel *i*. The decoded location is then taken as the pixel that maximizes $\log p\left( {\left\{ {n_j} \right\}_{j = 1}^N|i} \right)$. To take into account the dependence of the decoding error on the overall firing rate of the population, which differs across sessions, we subsampled between 17 and 25 neurons from all the active pyramidal cells for each time window in a 20-min session and computed the decoding error for each number of subsampled neurons. The dependence of the median decoding error as a function of the total number of spikes is shown in Fig. 4a. This dependence is exponential, with exponent that characterizes the decoding accuracy per spike.

### Computation of the pairwise correlation matrices

The cross-correlogram of spike trains of two neurons at time delay τ is computed as $ccg_{ij}\left( \tau \right) = \frac{1}{T}{\int}_0^T {f_i\left( t \right)f_j\left( {t + \tau } \right)dt}$, where *f*_*i*_(*t*) is the spike train of the *i*th neuron, and *T* is the total duration of the recording considered. Then, the correlation of firing of two neurons on a time scale of *τ*_*max*_ is computed as: $C_{ij} = \frac{1}{{\tau _{max}r_ir_j}}\max \left( {{\int}_0^{\tau _{max}} {ccg_{ij}\left( \tau \right)d\tau } ,\,{\int}_0^{\tau _{max}} {ccg_{ji}\left( \tau \right)d\tau } } \right)$, where *r*_*i*_ is the average firing rate of the *i*th neuron during the recording time considered. Notice that the resulting correlation value is always non-negative. This computation replicates Giusti et al.^16^, which developed the clique topology method to be used for analyzing the pairwise correlation matrix. For all figures shown, we used *τ*_*max*_ = 1 second. For all datasets, only neurons with average firing rates in the range 0.1–7 Hz were included in the pairwise correlation computation. For datasets obtained from CRCNS, we used spike times from only the last two-thirds of the total duration of each session. Changing *τ*_*max*_ to 500 ms does not change our conclusions: (1) Betti curves of all the displayed datasets can be fitted with a 3D hyperbolic geometry (*P* > 0.05 for both curve integral and L1 distance for all three Betti curves); (2) low-dimensional (≤10D) Euclidean geometry still cannot explain the Betti curves that are used to falsify Euclidean geometry in Fig. 2e,h and Extended Data Figs. 3 and 4; and (3) hyperbolic radius increases proportionally to the logarithm of time that the animal had to explore it.

For time shifting the spike trains as controls for Betti curves, a random time lag is added to each neuron’s spike train with periodic boundary condition applied so that the firing statistics of single neurons are preserved. The time lags are different across neurons to destroy the pairwise correlation statistics.

### Clique topology method for finding underlying hidden geometry in neural population responses

A useful technique to determine the geometry in which neuronal responses reside is clique topology. This method detects structures in a pairwise similarity matrix (or negative distance matrix) of points that are (1) invariant to any monotonic linear or non-linear transformations of the entries of the similarity matrix, making it ideal to use for neuronal responses that are known to be distorted by various monotonic functions and (2) characteristic of the underlying geometry. Here, we use pairwise correlation between CA1 neuronal responses (see above) for the similarity matrix as *S*_*ij*_ = *C*_*ij*_, where *S*_*ij*_ is the similarity between responses of cells *i* and *j*, and *C*_*ij*_ is the correlation between two cells’ responses. This metric intuitively is reflective of degree of place field overlap between two neurons^15^. Provided with the symmetric pairwise correlation matrix, the algorithm passes the matrix through a step function of different thresholds to only keep those entries in the matrix above the threshold (Fig. 2b). Based on these values, a topological graph is created (Fig. 2c). This graph can then be characterized by its numbers of cycles (holes) in one, two or higher dimensions (called one cycle, two cycles, etc.), excluding those that arise from boundaries of higher-dimensional cliques. With high thresholds of the step function, the number of cycles will, in general, be low, as most nodes are not interconnected. With low thresholds, the number of cycles will also, in general, be low, as nodes form fully connected networks. These numbers of cycles in one, two or higher dimensions at a set threshold are called the 1st Betti number, 2nd Betti number and so on. Plotting them as a function over the entire density range *ρ* of links (from highest threshold to lowest threshold) gives the so-called 1st Betti curve *β*_1_ (*ρ*), 2nd Betti curve *β*_2_ (*ρ*) and so on. These Betti curves are very sensitive to similarity matrices produced from different geometries and, hence, can be used for identifying the underlying geometric structure of CA1 neural representation. For our datasets, we use the first three Betti curves to search for the underlying geometries of the data. The CliqueTop MATLAB package was obtained online from the original authors^16^.

The pairwise correlation matrices of CA1 neuronal responses that we used for Betti curve generations are computed as described above. From a correlation matrix, we can also define the distance matrix between neuronal responses as *D*_*ij*_ = −*C*_*ij*_, where *D*_*ij*_ is the distance between neurons *i* and *j*, and *C*_*ij*_ is the correlation between two neurons as defined above. This definition makes neurons that are more correlated with each other to have shorter distance between them, consistent with intuition. We note that, with the algorithm being invariant under any non-linear monotonic transformations, it can be easily shown that this definition of distance can be monotonically transformed to become a true distance metric. Notably, the relative ordering of all the entries of a certain distance matrix may not be realizable in some geometries no matter how the points are configured, but, in other geometries, it becomes possible. Thus, the tool can be used to test geometries for their ability to support a found correlation matrix by inspecting those invariant features, namely the Betti curves, *β*_*m*_(*ρ*), where *ρ* is the edge density from 0 to 1.

To determine the geometry of the neuronal responses, we screened two kinds of geometries: Euclidean geometry of different dimensionality and hyperbolic geometry (native model) with different dimensionality and radii. In each geometry, we sampled points (same number as number of used CA1 neurons) uniformly according to the geometric properties. In d-dimensional Euclidean geometry, the points were sampled uniformly in the d-dimensional unit cube. In d-dimensional hyperbolic model, *ζ* is set as 1 while the maximal radius *R*_max_ of the geometry was adjusted to different values (this is equivalent to fixed maximal radius *R*_max_ with changing curvature *ζ*). The points are then sampled uniformly to have uniformly distributed angles and their radii *r* ∈ [0,*R*_max_] following the distribution *ρ*(*r*) ~ sinh^(*d*−1)^(*r*), which is approximately proportional to *e*^(*d*−1)*r*^ when *r* ≫ 1. With points sampled, we then compute their pairwise distance matrices with their respective distance metric: Euclidean distance for points sampled from Euclidean geometry and hyperbolic distance for points sampled from hyperbolic geometry. The distance metric between two points in a hyperbolic geometry of constant curvature K = − *ζ*^2^ < 0, *ζ* > 0, is given by the hyperbolic law of cosines:

$$\cosh \left( {\zeta x} \right) = \cosh \left( {\zeta r} \right)\cosh \left( {\zeta r\prime } \right) - \sinh \left( {\zeta r} \right)\sinh \left( {\zeta r\prime } \right)\cos \Delta \theta$$

where *x* is the hyperbolic distance; *ζ* is set as 1 in our model; *r* and *r*′ are the radial distances of the two points from the origin; and ∆θ is the angle between them.

Considering that a small amount of noise may exist in the correlation between firing of two CA1 neurons due to the different trajectories taken by the animal, the stochastic nature of neuronal firing and other high-order cognitive processes, we also added i.i.d. multiplicative Gaussian noise to each entry of the pairwise distance matrices obtained for both Euclidean model and hyperbolic model before generating the Betti curves from them (no noise added to the experimental pairwise correlation matrices). Thus, the final distance matrices to be used for Betti curve analyses have entries $D_{ij} = D_{ij}^{geo} \cdot$ (1 + *ϵ*∙*N*(0,1)), where $D_{ij}^{geo}$ is the geometric distance computed based on the coordinates of the sampled points *i* and *j*, and *ϵ* is the noise level set to be 0.05 for all the analyses in this paper. To summarize, sampled points from different geometries give different Betti curves through (1) different distribution of points in these geometries and (2) different distance metrics used to compute the pairwise distances.

To determine whether a geometry underlies the correlation among CA1 neuronal responses, we compare the Betti curves generated from that geometry (by sampling points as described above) to the Betti curves generated from the pairwise correlation matrices of CA1 neurons (experimental Betti curves). Specifically, we uniformly sample points (same number as number of CA1 neurons) from the geometry, compute the pairwise distance matrix, use clique topology on the negative pairwise distance matrix (to correspond to correlation matrix) to generate the Betti curves and repeat 300 times. As a result, we have 300 × 3 Betti curves, as for each sampling we obtain the first three Betti curves (*β*_1_ (*ρ*), *β*_2_ (*ρ*) and *β*_3_ (*ρ*)), counting number of cycles in one, two and three dimensions, respectively. For each Betti curve *β*_*m*_ (*ρ*), we compute its integrated Betti value, defined as $\bar \beta _m = {\int}_0^1 {\beta _m\left( \rho \right)d\rho }$, where *ρ* is the edge density, and *β*_*m*_ (*ρ*) denotes the m-th Betti curve. The integrated Betti values computed from the experimental Betti curves can then be compared to those generated from sampling in the given geometry (model Betti curves). We report the *P* values of integrated Betti values as the two-tailed percentiles for where the experimental integrated Betti values fall within the sampling-generated distributions (of 300 samples), separately for *β*_1_ (*ρ*), *β*_2_ (*ρ*) and *β*_3_ (*ρ*). Besides integrated Betti values, we also tested for L1 distances. Specifically, for each geometry, we calculate the average model Betti curves $\beta _1^{{{{\mathrm{ave}}}}}\left( \rho \right)$, $\beta _2^{{{{\mathrm{ave}}}}}\left( \rho \right)$ and $\beta _3^{{{{\mathrm{ave}}}}}\left( \rho \right)$ by averaging over all 300 sampling-generated model Betti curves. Then, we compute the L1 distance between the experimental Betti curves (*β*_*m*_ (*ρ*), m = 1, 2, 3) and the averages: $l_m = {\int}_0^1 {\left| {\beta _m\left( \rho \right) - \beta _m^{ave}\left( \rho \right)} \right|} d\rho$. The *P* value of this L1 distance is the one-tailed percentile of itself in the distribution of L1 distances computed from each sampling-generated Betti curve and the average (% sampling that resulted in a larger or equal L1 distance to average than the experimental L1 distance). For visualization purposes only, the experimental Betti curves were smoothed with a kernel size of 1/50 total number of edge densities of each Betti curve (no smoothing was used for computing integrated Betti values and L1 distances).

If a geometry gives statistically similar Betti curves compared to the experimental Betti curves, then the geometry is viable in explaining the neural representation. On the contrary, if the model Betti curves are dissimilar to the experimental Betti curves, then the geometry under testing is not viable.

### Determination of optimal *R*_max_ or dimension of hyperbolic geometry with Betti curves

To determine the optimal *R*_max_ of the hyperbolic geometry that captures a correlation matrix in Figs. 2 and 3, we searched over *R*_max_ in {5:0.5:24}. For each *R*_max_, we calculate the *P* values for integrated Betti values and L1 distances of the first two Betti curves (the 3rd Betti curve was excluded as its integrated Betti value becomes unstable when number of neurons is small). Multiplying the four *P* values, we obtain the *P* value product of them. The optimal *R*_max_ is defined as the *R*_max_ that results in the maximum *P* value product out of all the *R*_max_ values that have been tested. For a recording session, we randomly sample 75% of active CA1 neurons, compute their pairwise correlation matrix, determine the optimal *R*_max_ as above and repeat 100 times to obtain a distribution of *R*_max_ for the recording session.

To quantify how well the experimental Betti curves are fitted by model Betti curves from hyperbolic geometry of different dimensions, we used χ^2^ statistics. Specifically, we first binned the Betti curves (*n*_*bins*_ = 10 for each curve) and then computed the square of the difference between the experimental Betti curves after binning and the mean of the model Betti curves (estimated with bootstrap of 500 repetitions) after binning, denoted *D*_*expm*_. We also computed the square of the difference between model curves from each sampling of points and the mean of the model curves and took their average, denoted *D*_*model*_. The χ^2^ statistic is defined as $\chi ^2 = \frac{1}{{n_{bins}}}\mathop {\sum}\nolimits_{bins} {\frac{{D_{expm}}}{{D_{model}}}}$, where the division is bin-wise. Note that, when this χ^2^ statistic is small, it means that the deviation of model curves from the experimental curves is small, which indicates that the experimental curves are well-explained by the model and vice versa. We also tested whether the dimension of hyperbolic geometry has an effect on this χ^2^ statistic with two-way ANOVA, which also controls for the effect of different animals and sessions.

### Bayesian estimator of curvature/hyperbolic radius from place field sizes

We used a Bayesian estimator on place field sizes, taking into account that we can only observe place fields within a range of sizes [*s*_*l*_,*s*_*u*_] due to the size of the entire experimental environment, the threshold for determination of place fields and grid sizes for computing rate maps. The probability to observe one place field size *s* given curvature *ζ* of the representation is given by:

7 $$P\left( {s|\zeta } \right) = \frac{1}{{Z\left( \zeta \right)}}\zeta e^{ - \zeta s}$$

when *s*_*l*_ < *s* < *s*_*u*_ and 0 otherwise, and

8 $$Z\left( \zeta \right) = {\int}_{s_l}^{s_u} {ds\zeta e^{ - \zeta s}}$$

Then, by Bayes’ theorem,

9 $$P\left( {\zeta |s_1, \cdots ,s_N} \right) = \frac{{\zeta ^N}}{{Z\left( \zeta \right)^N}}e^{ - \mathop {\sum}\nolimits_{n = 1}^N {\zeta s_n} }P\left( \zeta \right)$$

where *P*(*ζ*) is the prior on *ζ* which we used uniform distribution. Then, the estimate of *ζ* is the *ζ* that maximizes *P*(*ζ*|*s*_1_,⋯,*s*_*N*_).

### Simulation on the effect of undersampling on estimated *R*_max_

For this simulation, we fix the radius of the hyperbolic geometry to be 10 and randomly draw 41 points from it (same as the number of neurons in Fig. 2d–f). Then, at each time step of 1 second (same time scale as *τ*_max_ in the correlation calculation), we observe a noise-corrupted version of the pairwise distances (or negative pairwise correlations) among their responses. Specifically, we apply multiplicative Gaussian noise to the true pairwise distance matrix entrywise with *ϵ* = 0.5 in the formula above. At each readout time, we average all the observations so far to obtain one pairwise distance matrix to be used for Betti curve analysis on estimating the underlying *R*_max_, using the method just described above.

### Fisher information of model neurons

We modeled the neurons to have Gaussian tuning: $f_i\left( s \right) = A_iexp\left( { - \frac{1}{2}\left( {s - \mu _i} \right)^T\mathop {\sum}\nolimits_i^{ - 1} {\left( {s - \mu _i} \right)} } \right)$, with stimulus *s* = [*x*, *y*] as a 2D random variable taking values uniformly from [0, 1] × [0, 1]. For each neuron, we sample *A*_*i*_ ∼ *unif* [5,25], *μ*_*i*_ ∼ *unif*([0, 1] × [0, 1]) and Σ_*i*_ = R(*θ*_*i*_)diag($\sigma _{i1}^2,\sigma _{i2}^2$)R(*θ*_*i*_)^T^, where R(*θ*_*i*_) is the rotation matrix $$\left( {\begin{array}{*{20}{c}} {\cos \theta _i} & { - \sin \theta _i} \\ {\sin \theta _i} & {\cos \theta _i} \end{array}} \right)$$ with *θ*_*i*_ ∼ *unif* [0, 2*π*], and *σ*_*i*1_ and *σ*_*i*2_ are i.i.d. ∼ exp(−*ζσ*), with *ζ* denoting the curvature of the representation geometry, which dictates the decay rate of the exponential distribution, and *ζ*^−1^ is the mean of the distribution. For uniform distribution of place field sizes, *σ*_*i*1_ and *σ*_*i*2_ are i.i.d. ∼ *unif* [0, 2*ζ*^−1^] to keep the same mean as in the exponential case.

We also assume the neurons to have independent Poisson variability^48^. Thus, given a stimulus *s*, the number of spikes fired by a neuron is *r*_*i*_|*s* ∼ *Poisson*(*f*_*i*_ (*s*)). These neurons’ responses then encode a posterior distribution on *s*: *p*(*s*|*r*_1_,⋯,*r*_*N*_). For each of 10,000 iterations, a stimulus *s* is randomly generated ∼ *unif*([0, 1] × [0, 1]) with *A*_*i*_, *μ*_*i*_ and Σ_*i*_ all randomly generated again with the above distributions. The Fisher information matrix *I*(*s*) is computed by definition:

10 $$I\left( s \right)_{i,j} = E\left[ {\left( {\frac{\partial }{{\partial s_i}}\log p\left( {r_1, \cdots ,r_N|s} \right)} \right)\left( {\frac{\partial }{{\partial s_j}}\log p\left( {r_1, \cdots ,r_N|s} \right)} \right)|s} \right]$$

which, for the independent neuron model as we assume here, simplifies to:

11 $$I\left( s \right)_{i,j} = - \mathop {\sum}\nolimits_{i = 1}^N E \left[ {\frac{{\partial ^2}}{{\partial s_i\partial s_j}}\log p\left( {r_i|s} \right)|s} \right]$$

So, with Fisher information matrix *I*(*s*) computed each time, we can compute the average magnitude of the 10,000 Fisher information matrices, defined as det(*I*(*s*))^0.5^. Second-order polynomial is used for fittings, and the optimal size of the hyperbolic representation is determined as the *R*_max_ under the peak of the fitting polynomial.

For the case of each cell having potentially multiple place fields, we extracted the number of place fields from three 20-min sections in Fig. 3. Dividing them by the number of all cells gives mean number of place fields per cell. Similarly, we can compute standard deviations of the number of place fields across cells. The number of place fields per cell is experimentally described by a gamma distribution^20^, whose scale and shape parameters can now be determined by the average values of means and standard deviations computed from the three sessions. After drawing the number of place fields for each of *N* cells, the number is rounded to the closest integer. Then, for each place field, *A*_*i*_ and Σ_*i*_ are randomly generated as above, and the Fisher information matrix can be computed for a random *s*. To also match the size of the environment to the experiment (1.8 × 1.8 m), *μ*_*i*_ is now drawn from *unif*([0, 1.8] × [0, 1.8]) and so is *s* for each of 10,000 iterations.

### Statistics and reproducibility

No statistical method was used to predetermine sample size, but our sample sizes are similar to those reported in previous publications^12,16,30,41^. We analyzed data from previous publications^20,23,24^. For rats exploring the 48-m linear track, two rats had fewer than 30 active neurons (overall average firing rate within the range 0.1–7 Hz) recorded, and they were excluded from further analyses. For rats exploring a 180 × 180-cm box, all sessions that have more than 50 simultaneously recorded CA1 neurons were included for analysis. We also excluded neurons that are marked as inhibitory or not identified and those that have average firing rates outside the range 0.1–7 Hz during the entire recording session. We also excluded neurons that have average firing rates between 0.1 Hz and 7 Hz but remain silent (fire zero spikes) for more than 30 min for potential death of the cell or movement of electrodes. Details of sessions used are summarized in Supplementary Table 2. There was no randomization or division into experimental groups. Analyses were not performed blinded to the conditions of the experiments. Throughout the study, we employed non-parametric statistical methods. For comparisons between two groups, we used unpaired two-sample *t*-tests. Data distribution was assumed to be normal, but this was not formally tested. For comparisons among more than two groups, ANOVA with Tukey’s post hoc test was used. For checking whether samples are likely coming from a specific theoretical distribution, χ^2^ GOF test was used. Correlations are reported using Pearson’s correlation coefficient. More information can be found in the Nature Research Reporting Summary.

### Reporting summary

Further information on research design is available in the Nature Portfolio Reporting Summary linked to this article.

## Online content

Any methods, additional references, Nature Portfolio reporting summaries, source data, extended data, supplementary information, acknowledgements, peer review information; details of author contributions and competing interests; and statements of data and code availability are available at 10.1038/s41593-022-01212-4.

## Supplementary information

Supplementary Information Supplementary Tables 1 and 2 and Supplementary Figs. 1 and 2.

Reporting Summary

## Data Availability

All datasets used in this study, except rats running on the 48-m linear track, were generously contributed by György Buzsáki at http://crcns.org/data-sets/hc/hc-3 (refs. ^23,24^). See Supplementary Table 2 for the sessions used. The datasets in which rats ran on the 48-m linear track are made available on https://crcns.org/data-sets/hc/hc-31/. Source data are provided with this paper.

## Source data

Source Data Fig. 1 Statistical Source Data
Source Data Fig. 2 Statistical Source Data
Source Data Fig. 3 Statistical Source Data
Source Data Fig. 4 Statistical Source Data
Source Data Fig. 5 Statistical Source Data
Source Data Fig. 6 Statistical Source Data
Source Data Extended Data Fig. 1 Statistical Source Data
Source Data Extended Data Fig. 2 Statistical Source Data
Source Data Extended Data Fig. 3 Statistical Source Data
Source Data Extended Data Fig. 4 Statistical Source Data
Source Data Extended Data Fig. 5 Statistical Source Data
Source Data Extended Data Fig. 6 Statistical Source Data
Source Data Extended Data Fig. 7 Statistical Source Data
Source Data Extended Data Fig. 8 Statistical Source Data
Source Data Extended Data Fig. 9 Statistical Source Data
Source Data Extended Data Fig. 10 Statistical Source Data

## Code availability

Code written in MATLAB 2018b to simulate Fisher information models is publicly accessible on GitHub at https://github.com/HuanqiuZhang/Fisher_info_codes. The CliqueTop MATLAB package (2015 version) used for computing Betti curves is obtained from the original authors^16^ at https://github.com/nebneuron/clique-top. Other codes are available upon reasonable request.
