# Supplementary material for: Hippocampal spatial representations exhibit a hyperbolic geometry that expands with experience
Source: Nat Neurosci. 2022 Dec 29;26(1):131–9. doi: 10.1038/s41593-022-01212-4 (PMC9829541; doi:10.1038/s41593-022-01212-4)
Supplement: Supplementary file 1 — Supplementary Tables 1 and 2 and Supplementary Figs. 1 and 2. [file 41593_2022_1212_MOESM1_ESM.pdf]

nature neuroscience

Article https://doi.org/10.1038/s41593-022-01212-4

# Hippocampal spatial representations exhibit a hyperbolic geometry that expands with experience

In the format provided by the authors and unedited

| Animal#/ sessionID | hyp rad | hyp IBV-1 | hyp IBV-2 | hyp IBV-3 | hyp L1-1 | hyp L1-2 | hyp L1-3 | Euc IBV-1 | Euc IBV-2 | Euc IBV-3 | Euc L1-1 | Euc L1-2 | Euc L1-3 |
|---|---|---|---|---|---|---|---|---|---|---|---|---|---|
| animal1 | 15.5 | 0.780 | 0.873 | 0.833 | 0.960 | 0.920 | 0.823 | 0 | 0 | 0 | 0 | 0 | 0 |
| animal2 | 15 | 0.960 | 0.827 | 0.860 | 0.757 | 0.683 | 0.960 | 0 | 0 | 0 | 0 | 0 | 0 |
| animal3 | 13 | 0.727 | 0.973 | 0.820 | 0.417 | 0.497 | 0.687 | 0 | 0 | 0 | 0 | 0 | 0 |
| ec014.468 | 10.5 | 0.720 | 0.993 | 0.893 | 0.457 | 0.863 | 0.870 | 0 | 0 | 0 | 0 | 0 | 0 |
| ec014.639 | 12.5 | 0.213 | 0.947 | 0.493 | 0.287 | 0.790 | 0.233 | 0.007 | 0 | 0 | 0.003 | 0 | 0 |
| ec014.215 | 11.5 | 0.673 | 0.840 | 0.807 | 0.677 | 0.597 | 0.923 | 0 | 0 | 0 | 0 | 0 | 0 |
| ec014.260 | 11.5 | 0.960 | 0.833 | 0.967 | 0.917 | 0.797 | 0.963 | 0 | 0 | 0 | 0 | 0 | 0 |
| ec014.277 | 13.5 | 0.767 | 0.993 | 0.920 | 0.597 | 0.677 | 0.563 | 0 | 0 | 0 | 0 | 0 | 0 |
| ec014.333 | 13.5 | 0.807 | 0.540 | 0.893 | 0.623 | 0.817 | 0.237 | 0 | 0 | 0 | 0 | 0 | 0 |
| ec016.397 | 12.5 | 0.953 | 0.933 | 0.667 | 0.697 | 0.950 | 0.857 | 0 | 0 | 0 | 0 | 0 | 0 |
| ec016.582 | 14 | 0.827 | 0.427 | 0.513 | 0.927 | 0.697 | 0.750 | 0 | 0 | 0 | 0 | 0 | 0 |
| i01-maze15-MS.001 | 11.5 | 0.840 | 0.820 | 0.707 | 0.620 | 0.410 | 0.750 | 0 | 0 | 0 | 0 | 0 | 0 |

Table S1: **Radii used for 3D hyperbolic model and the fitting statistics for all sessions.** Each number from the 3rd column on is the *p*-value of the corresponding experimental value among the distribution of 300 corresponding model values (two-sided for integrated Betti values and one-sided for L1 distances). There are six experimental values for each session: integrated Betti values (IBV) for Betti 1, 2 and 3, and L1 distances for Betti 1, 2 and 3. Columns 3 to 8 are *p*-values in 3D hyperbolic geometry, and columns 9 to 14 are *p*-values in 3D Euclidean geometry.

| Square box datasets: | | | | | | |
|---|---|---|---|---|---|---|
| Session ID | Duration (sec) | Familiarity | Total # CA1 pyramidal neurons recorded | # active neurons (0.1 - 7 Hz) used for correlation matrix | Time interval used for correlation matrix (min) | Average firing rate of active neurons in the interval |
| ec014.215 | 5598 | 1 | 77 | 56 | [30 90] | 0.8783 |
| ec014.260 | 5494 | 3 | 52 | 39 | [30 90] | 0.5676 |
| ec014.277 | 5514 | 4 | 74 | 48 | [30 90] | 0.6777 |
| ec014.333 | 5609 | 7 | 54 | 29 | [30 90] | 0.4227 |
| ec016.397 | 5450 | 6 | 51 | 27 | [30 90] | 0.7303 |
| ec016.582 | 5452 | 10 | 81 | 42 | [30 90] | 0.5472 |
| i01-maze15-MS.001 | 3719 | 10 | 82 | 33 | [30 60] | 0.8444 |
| | | | | | | |
| Linear track (250cm) datasets: | | | | | | |
| ec014.468 | 3958 | 1 | 71 | 53 | [20 60] | 0.9390 |
| ec014.639 | 3879 | 8 | 61 | 24 | [20 60] | 0.8749 |

Table S2: **Details of sessions obtained from http://crcns.org/data-sets/hc/hc-3 for analyses.** Due to incompleteness of data, we assumed the animal ec014 was exposed to the square box for 90 min during each of its 2nd, 5th and 6th exposures.

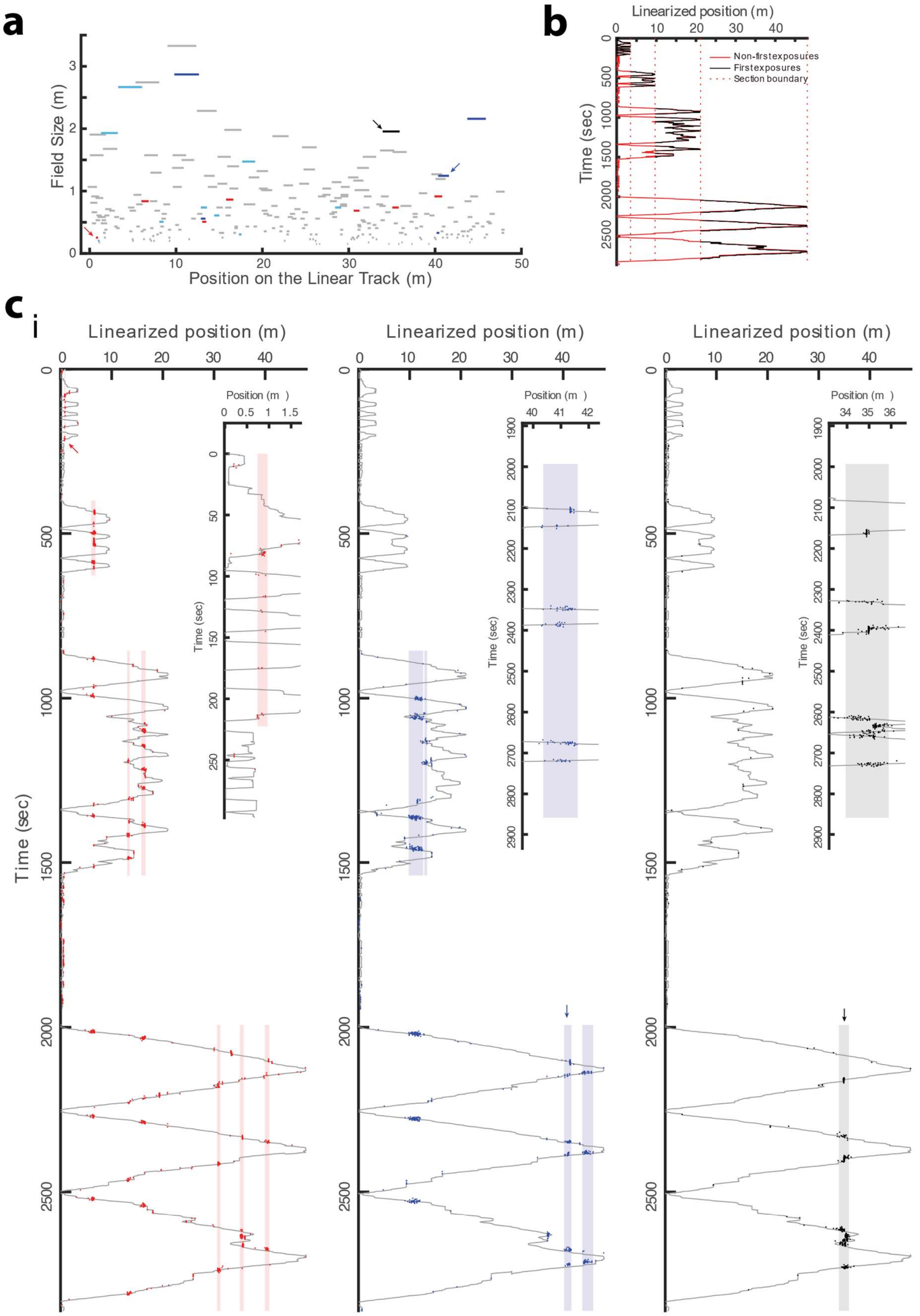
a
Field Size (m)
Position on the Linear Track (m)
b
Linearized position (m)
Time (sec)
Non-firstexposures
Firstexposures
Section boundary
c
i
Linearized position (m)
Time (sec)
Position (m )
Time (sec)
Linearized position (m)
Position (m )
Time (sec)
Linearized position (m)
Position (m )
Time (sec)

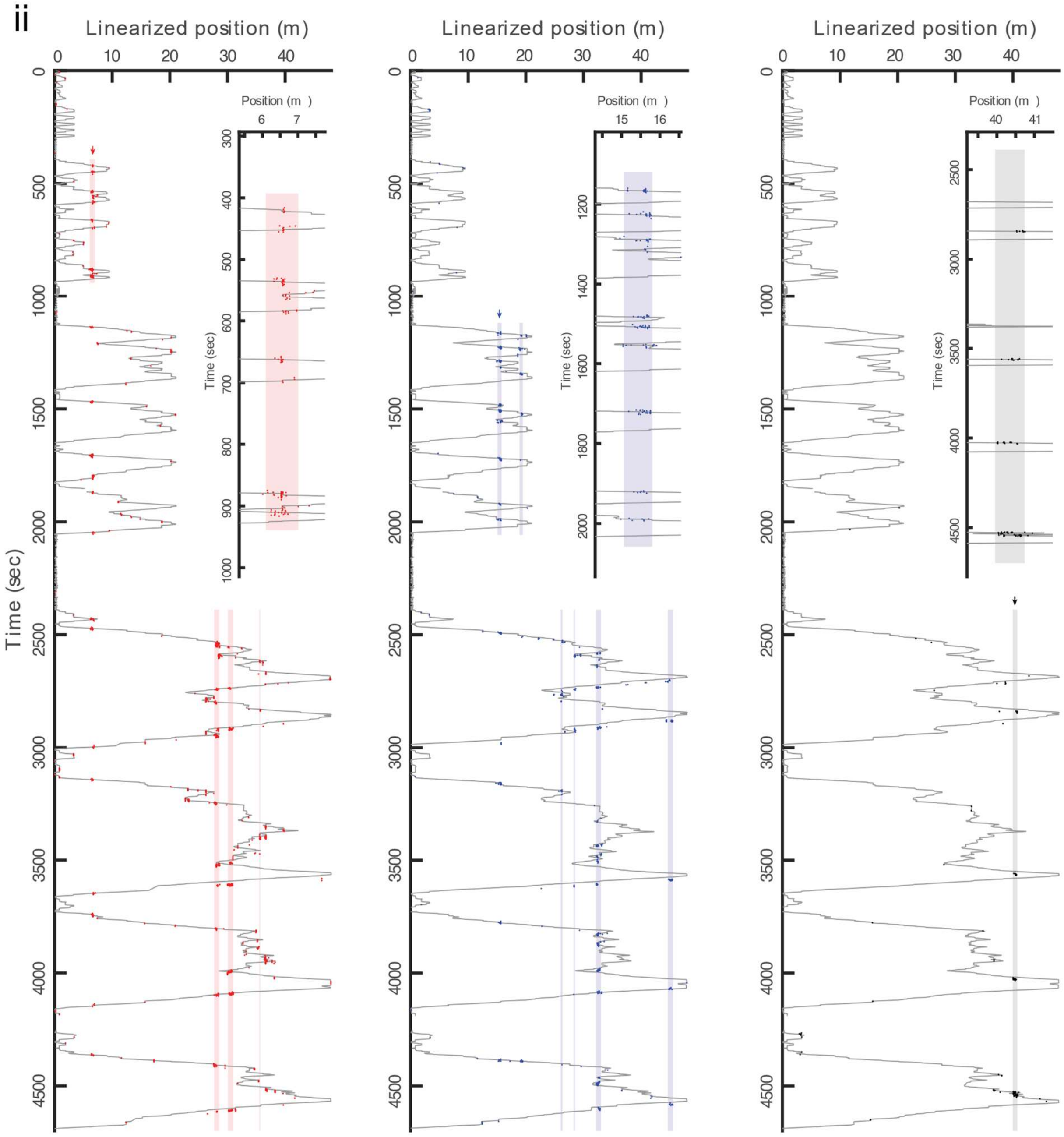

ii
Linearized position (m)
Linearized position (m)
Linearized position (m)
Time (sec)
Position (m )
Position (m )
Position (m )
Time (sec)
Time (sec)
Time (sec)

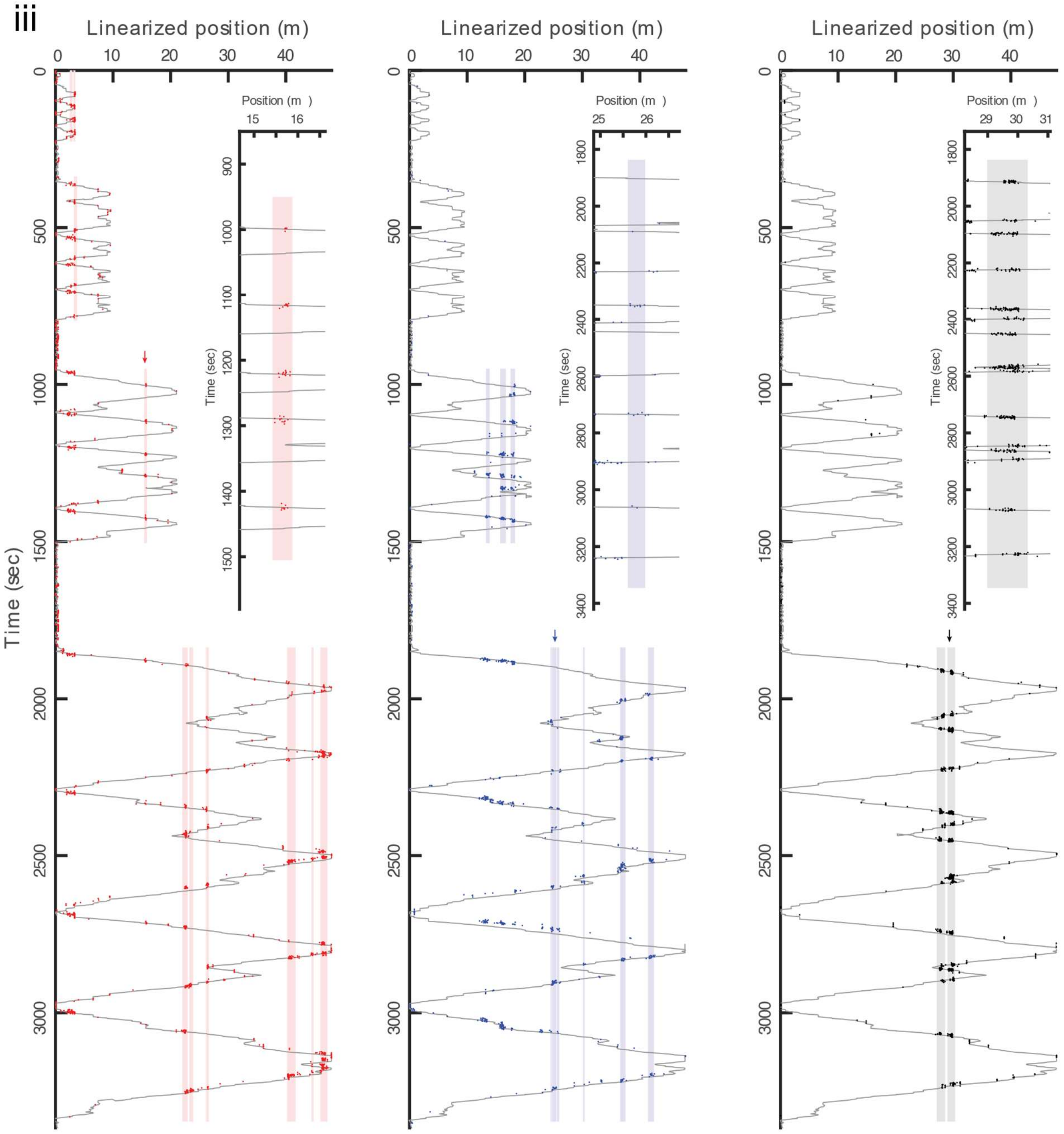

iii
Linearized position (m)
Linearized position (m)
Linearized position (m)
Time (sec)
Position (m )
Time (sec)
Position (m )
Time (sec)
Position (m )
Time (sec)

Supplementary Figure 1: **Spatial firing of individual cells from the 48-meter linear track.** (**a**) Same plot as in Fig. 1c with color code for fields from 3 different cells shown in **c**(i). Arrows indicate specific place fields zoomed-in in the insets of **c**(i). (**b**) Linearized trajectory of one animal through the linear track over time. Dashed red lines indicate the section boundaries of the linear track which is extended after each epoch (Fig. 3c). Black lines indicate periods when the animal is exposed to the corresponding section for the 1st time. Red lines indicate non-first exposures. (**c**) Spatial firing of three individual cells from each of the three animals (i-iii). Each column shows the position of the animal over time (gray line) and all spikes (dots) from one cell. Vertical jitter has been added to spikes to aid visualization. The spatial extent of detected place fields is shown as colored bars. Arrows indicate the place fields zoomed-in in the insets. Place fields were detected independently for each of the four epochs and only those formed within first exposure periods (black lines in **b**) were considered in the analyses.

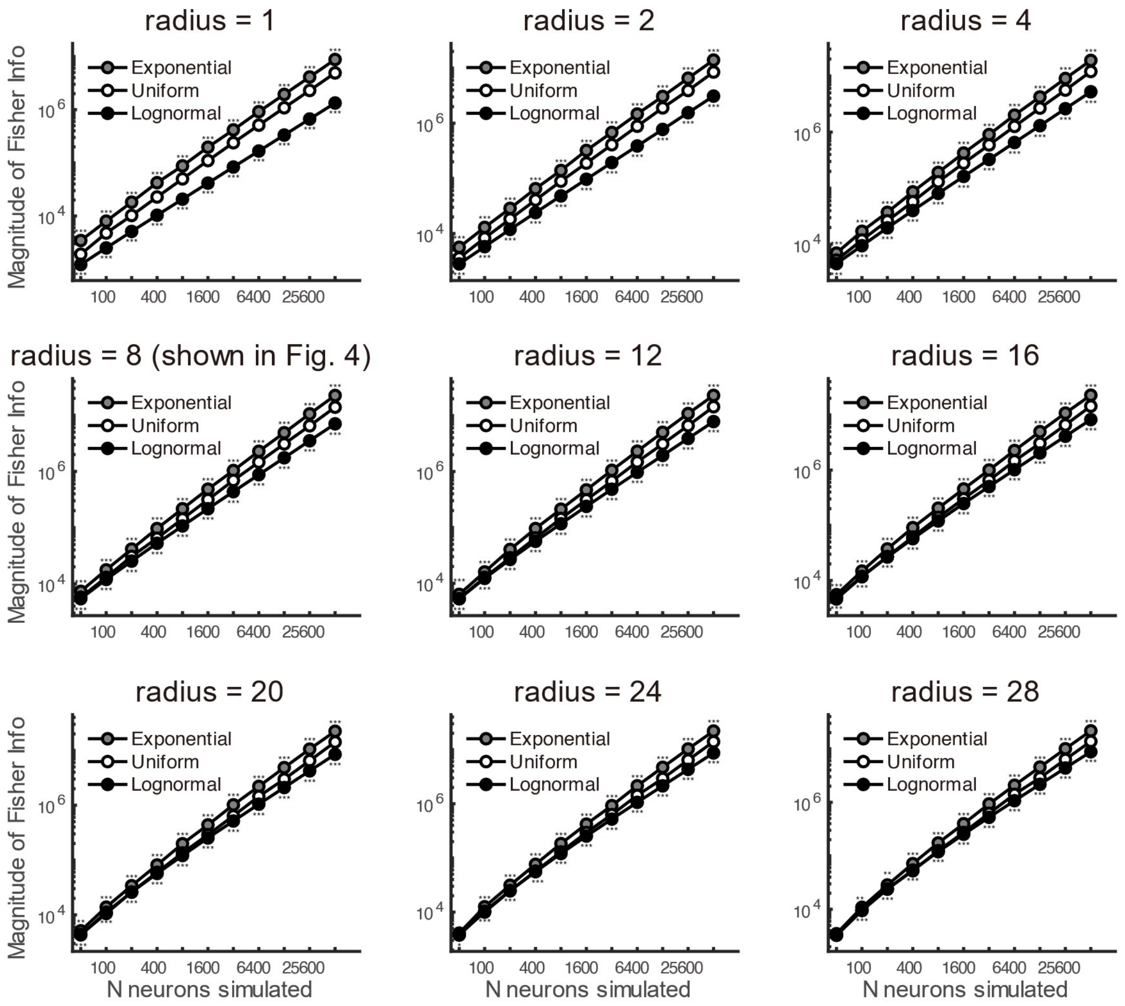


Supplementary Figure 2: **Comparison of magnitude of Fisher information provided when $\sigma$ is exponentially distributed vs. uniformly distributed and lognormally distributed.** For each panel, a different value of hyperbolic radius is used (shown at the top of each panel) for sampling $\sigma$. In each panel, the mean of the uniform distribution is matched to the mean of the exponential distribution, and both the mean and variance of the lognormal distribution are matched to the mean and variance of the exponential distribution. At each number of neurons simulated, unpaired two-sample two-sided t-tests are performed for the same mean information between exponential and uniform distributions (results shown as asterisks above the exponential values), and between exponential and lognormal distributions (results shown as asterisks below the lognormal values). $p$-value≤0.05:*; $p$-value≤0.01:**; $p$-value≤0.001:***.
