# Supplementary material for: Hippocampal spatial representations exhibit a hyperbolic geometry that expands with experience
Source: Nat Neurosci. 2022 Dec 29;26(1):131–9. doi: 10.1038/s41593-022-01212-4 (PMC9829541; doi:10.1038/s41593-022-01212-4)
Supplement: Supplementary file 2 — Reporting Summary [file 41593_2022_1212_MOESM2_ESM.pdf]

nature portfolio

Corresponding author(s): Tatyana Sharpee

Last updated by author(s): Oct 13, 2022

Reporting Summary

Nature Portfolio wishes to improve the reproducibility of the work that we publish. This form provides structure for consistency and transparency in reporting. For further information on Nature Portfolio policies, see our Editorial Policies and the Editorial Policy Checklist.

Statistics

For all statistical analyses, confirm that the following items are present in the figure legend, table legend, main text, or Methods section.

n/a	Confirmed	
☐	☒	The exact sample size (*n*) for each experimental group/condition, given as a discrete number and unit of measurement
☐	☒	A statement on whether measurements were taken from distinct samples or whether the same sample was measured repeatedly
☐	☒	The statistical test(s) used AND whether they are one- or two-sided<br>*Only common tests should be described solely by name; describe more complex techniques in the Methods section.*
☒	☐	A description of all covariates tested
☐	☒	A description of any assumptions or corrections, such as tests of normality and adjustment for multiple comparisons
☐	☒	A full description of the statistical parameters including central tendency (e.g. means) or other basic estimates (e.g. regression coefficient) AND variation (e.g. standard deviation) or associated estimates of uncertainty (e.g. confidence intervals)
☐	☒	For null hypothesis testing, the test statistic (e.g. *F*, *t*, *r*) with confidence intervals, effect sizes, degrees of freedom and *P* value noted<br>*Give P values as exact values whenever suitable.*
☐	☒	For Bayesian analysis, information on the choice of priors and Markov chain Monte Carlo settings
☒	☐	For hierarchical and complex designs, identification of the appropriate level for tests and full reporting of outcomes
☐	☒	Estimates of effect sizes (e.g. Cohen's *d*, Pearson's *r*), indicating how they were calculated

Our web collection on statistics for biologists contains articles on many of the points above.

Software and code

Policy information about availability of computer code

Data collection	No software was used for data collection as all the experimental data were obtained from published sources.
Data analysis	Code written in Matlab 2018b to simulate Fisher information models is publicly accessible on GitHub at https://github.com/HuanqiuZhang/Fisher_info_codes. The CliqueTop Matlab package (2015 version) used for computing Betti curves is obtained from the original authors (ref. 16) at https://github.com/nebneuron/clique-top. Other codes are available upon request.

For manuscripts utilizing custom algorithms or software that are central to the research but not yet described in published literature, software must be made available to editors and reviewers. We strongly encourage code deposition in a community repository (e.g. GitHub). See the Nature Portfolio guidelines for submitting code & software for further information.

Data

Policy information about availability of data

All manuscripts must include a data availability statement. This statement should provide the following information, where applicable:

- Accession codes, unique identifiers, or web links for publicly available datasets
- A description of any restrictions on data availability
- For clinical datasets or third party data, please ensure that the statement adheres to our policy

All datasets used in this study except rats running on the 48-meter linear track are generously contributed by Dr. Buzsaki at http://crcns.org/data-sets/hc/hc-3. See Table S2 for the sessions used.
The datasets in which rats ran on the 48-meter linear track will be made available on crcns.org.

March 2021

Field-specific reporting

Please select the one below that is the best fit for your research. If you are not sure, read the appropriate sections before making your selection.

☒ Life sciences ☐ Behavioural & social sciences ☐ Ecological, evolutionary & environmental sciences

For a reference copy of the document with all sections, see nature.com/documents/nr-reporting-summary-flat.pdf

Life sciences study design

All studies must disclose on these points even when the disclosure is negative.

Sample size	No statistical method was used to predetermine sample size, but our sample sizes are similar to those reported in previous publications (refs. 12,16,30,41).
Data exclusions	For rats exploring the 48-meter linear track, there were two rats with less than 30 active neurons (overall average firing rate within the range 0.1 - 7 Hz) recorded, which are excluded from further analyses. For rats exploring a 180 x 180 cm box, all sessions that have more than 50 simultaneously recorded CA1 neurons were included for analysis. We also excluded neurons that are marked as inhibitory or not identified, and those that have average firing rates outside the range 0.1 - 7 Hz during the entire recording session. We also excluded neurons that have average firing rates between 0.1 and 7 Hz, but remain silent (fire 0 spike) for more than 30 min for potential death of the cell or movement of electrodes.
Replication	The main findings presented in this study were consistently replicated across 5 animals, environment types (5 linear sessions and 7 square sessions), and environment sizes (3 sessions on 48-meter linear track and 2 sessions on 2.5-meter linear track).
Randomization	There was no randomization or division into experimental groups.
Blinding	Analyses were not performed blinded to the conditions of the experiments.

Reporting for specific materials, systems and methods

We require information from authors about some types of materials, experimental systems and methods used in many studies. Here, indicate whether each material, system or method listed is relevant to your study. If you are not sure if a list item applies to your research, read the appropriate section before selecting a response.

Materials & experimental systems

n/a	Involved in the study	
☒	☐	Antibodies
☒	☐	Eukaryotic cell lines
☒	☐	Palaeontology and archaeology
☒	☐	Animals and other organisms
☒	☐	Human research participants
☒	☐	Clinical data
☒	☐	Dual use research of concern

Methods

n/a	Involved in the study	
☒	☐	ChIP-seq
☒	☐	Flow cytometry
☒	☐	MRI-based neuroimaging
